# 3D printing combined with thermally induced phase separation for engineering hierarchical osteogenic PLA scaffolds

**DOI:** 10.1016/j.mtbio.2025.102621

**Published:** 2025-12-01

**Authors:** Xinyi Yun, Ziyue Li, Zi Yan, Shiyu Li, Zhenning Dai, Jintao Hu, Yueyi Ren, Liming Huang, Qingshi Wang, Chengyu Zhang, Jianxin Li, Chunnuan Deng, Han Liu, Weihan Zheng, Chong Zhong, Ziqi Zhang

**Affiliations:** a Department of Biliary-Pancreatic Surgery, State Key Laboratory of Traditional Chinese Medicine Syndrome, The First Affiliated Hospital of Guangzhou University of Chinese Medicine, Guangzhou University of Chinese Medicine, Guangzhou, 510405, China; b Department of Immunology, Institute of Geriatric Immunology, School of Medicine, Jinan University, Guangzhou, 510632, China; c Department of Cardiovascular Medicine, The Second Xiangya Hospital, Central South University, Changsha, 410000, China; d Department of Urology, Sun Yat-sen Memorial Hospital, Sun Yat-sen University, Guangzhou, 510120, China; e Institute of Translational Medicine, Shanghai University, Shanghai, 200444, China; f Sanming Second Hospital, Sanming, 366000, China; g Department of Stomatology, Guangdong Provincial Key Laboratory of Research and Development in Traditional Chinese Medicine, Guangdong Second Traditional Chinese Medicine Hospital, Guangzhou, 510095, China; h Angiitis Department of The Affiliated Traditional Chinese Medicine Hospital, Guangzhou Medical University, Guangzhou, 51006, China; i Guangdong Medical Innovation Platform for Translation of 3D Printing Application, The Third Affiliated Hospital of Southern Medical University, Southern Medical University Guangzhou, 510630, China

**Keywords:** 3D printing, Thermally induced phase separation, Polylactic acid, Bone repair, Micro-nano fibrous structure

## Abstract

Accelerated population aging and rising incidence of bone defects have intensified the need for advanced bone regeneration strategies. While tissue-engineered scaffolds fabricated via 3D printing offer promising alternatives to conventional grafts, most techniques fail to replicate the multi-scale fibrous architecture of native bone extracellular matrix, limiting their biofunctionality. To address this, we developed a hybrid manufacturing strategy integrating low-temperature thermally induced phase separation with extrusion-based 3D printing of polylactic acid (PLA) scaffolds. By optimizing solvent ratios (THF: DMF = 3:1) and freezing temperatures (−196 °C–4 °C), we produced scaffolds with tunable micro-nano fibrous surfaces and macroporous structures. Key findings revealed that scaffolds processed at −196 °C (PLA-196) exhibited the highest porosity (pore size: 6.01 ± 2.06 μm), superior hydrophilicity, and enhanced compressive modulus. These scaffolds significantly promoted BMSC adhesion, proliferation, and osteogenic differentiation via activation of *Macf1*-mediated pro-osteogenic signaling pathways, leading to elevated expression of Runx2, Ocn, and Col-I. *In vivo*, PLA-196 scaffolds accelerated cranial defect healing, achieving 62 % bone volume fraction and robust vascularization within 8 weeks. This study demonstrates a scalable, biocompatible platform for fabricating hierarchically structured bone scaffolds that bridge the gap between structural complexity and biological efficacy, offering significant potential for clinical translation in regenerative medicine.

## Graphical abstract

**Figure:**
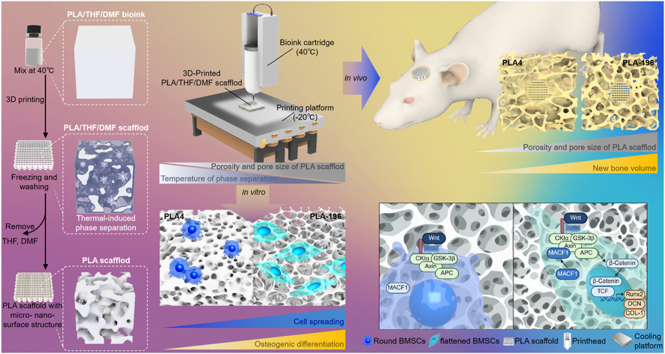

## 1 Introduction

Accelerated population aging, along with factors like trauma and tumors, has led to a rising incidence of bone defects. While autografts, allografts, and metal implants—the conventional treatment options—all exhibit significant limitations, tissue-engineered bone has emerged as a promising alternative [1,2]. The advancement of 3D printing technology offers new opportunities for bone repair by enabling the fabrication of personalized scaffolds that mimic anatomical contours and provide mechanical support [3,4]. These scaffolds facilitate bone in growth through their macroporous structures [5]. However, conventional extrusion- or photopolymerization-based 3D printing techniques are unable to replicate the micro- and nanoscale fibrous architectures of the natural extracellular matrix. To address this, several advanced strategies have been developed. For instance, hierarchical porosity encompassing both macro- and micro-scale pores has been achieved through composite sacrificial templates, such as incorporating water-soluble polyvinyl alcohol and leachable salts (e.g., NaCl) into polymer matrices, followed by post-printing leaching to create interconnected microporous networks [5,6]. Additionally, the integration of electrospun nanofibers (e.g., polyacrylonitrile) onto 3D-printed scaffolds has been used to impart nanotextured surfaces that enhance cell adhesion and proliferation [7]. Novel structural designs, such as dual-pore Kagome architectures, have also been proposed to simultaneously improve mechanical properties and biological responses [8]. Furthermore, the use of nanofillers like cellulose nanocrystals has been shown to reduce porosity and improve geometric accuracy in printed polyhydroxyalkanoate-based scaffolds [9]. However, these 3D printing strategies involve complex composite material systems or intricate processing systems [10], resulting in longer preparation workflows and unstable fabrication outcomes.

In the context of advancing 3D printing technologies for bone tissue engineering, phase separation techniques combined with photo-curing methods have shown significant potential for creating scaffolds with multi-scale architectures [11,12]. For instance, Paquet et al. [13] utilized a photosensitive resin composite with silver ions to achieve submicron-scale porosity on macrostructures through 3D printing, demonstrating an approach to enhance surface morphology for potential antimicrobial and structural benefits. Similarly, the Levkin team [14] employed a system comprising hydroxyethyl methacrylate, ethylene glycol dimethacrylate, cyclohexanol, decanol, and i819 photoinitiator to realize digital light processing 3D printing integrated with polymerization-induced phase separation, resulting in porous scaffolds with nanofibrous surface structures [15]. These studies highlight the progress in combining phase separation with light-based 3D printing to achieve intricate micro- and nano-features, although challenges such as bio-toxicity from certain bioinks remain a concern for bone repair applications [[16], [17], [18], [19]]. This background underscores the need for further innovation in material systems and printing strategies to improve biocompatibility and functional performance in bone defect regeneration.

In contrast, traditional 3D printing materials for bone repair, such as polylactic acid (PLA), offer advantages like FDA-approved biodegradability, good biocompatibility, tunable degradation rates, and favorable mechanical properties [20]. PLA's degradation products, lactic acid, are metabolized into carbon dioxide and water without systemic toxicity, and it can be blended with other materials to modify its characteristics. Despite these benefits, conventional 3D printing techniques for PLA, such as fused deposition modeling, struggle to achieve an integrated construction of macroporous scaffolds with micro-fibrous surfaces in a simple material system [12,[21], [22], [23]]. This is because these methods often lack the precision to control micro-scale features simultaneously with macro-porosity, limiting their effectiveness in promoting cell infiltration and tissue regeneration.

The incorporation of micro/nanofibrous architectures onto scaffold surfaces significantly enhances bone regeneration by closely mimicking the native extracellular matrix topology, which provides optimal attachment sites to promote cell adhesion, spreading, migration, and proliferation [24,25]. Furthermore, these fibrous structures activate critical pro-osteogenic signaling pathways, including Wnt/β-catenin, PI3K/Akt, and mTOR, largely mediated through cytoskeletal regulator MACF1, which stabilizes β-catenin signaling and enhances osteoblast differentiation and bone formation [26,27]. This combined structural and biochemical mimicry synergistically improves cellular responses essential for effective bone regeneration [28].

To overcome these challenges, this study proposes a novel hybrid manufacturing strategy that integrates phase separation principles with 3D printing. Through extensive experimentation with varying material ratios and phase separation temperature conditions, it was observed that low temperatures significantly enhance the migration and crystallization rates of polymer components in the solution. This temperature-induced effect slows polymer chain movement, increases viscosity, and promotes rapid solidification and aggregation, ultimately facilitating the production of 3D printed PLA scaffolds with a micro-nano fibrous morphology. These scaffolds exhibit optimal porosity and pore size distributions, conducive to cell growth and bone repair, thereby addressing the gap in existing technologies.

## 2 Methods

### 2.1 Preparation and printing of composite materials

The homogeneous PLA solution formulated in tetrahydrofuran (THF)/N, N-dimethylformamide (DMF) co-solvent system served as the bioink for 3D-printing. Under the condition of a constant proportion of 30 % (w/v) PLA, bioink were grouped according to the ratio of THF and DMF (v/v): 1:1, 2:1, 3:1, 4:1, 5:1 as in Table 1.

**Table 1:** Proportion of solvents in different polymeric ink.

Group/Ratio	PLA (g)	THF (mL)	DMF (mL)
PLA-1:1	1.50	2.50	2.50
PLA-2:1	1.50	3.33	1.67
PLA-3:1	1.50	3.75	1.25
PLA-4:1	1.50	4.00	1.00
PLA-5:1	1.50	4.17	0.83

For example, the configuration of the 3:1 group bioink was as fellow: 1.5 g of PLA powder (Jinan Daigang Biological Engineering Co., Ltd., China) was dissolved in 5 mL of the mixed solvent containing 3.75 mL THF (Guangzhou Dongzheng Chemical Plant Co., Ltd., China) and 1.25 mL DMF (Guangzhou Dongzheng Chemical Plant Co., Ltd., China). The mixture was continuously agitated using a magnetic stirrer at 40 °C for 2 h to ensure complete dissolution of the bioink. The dissolved PLA solution was removed from the magnetic stirrer, loaded into the bioink cartridge of a 3D bioprinter (3D-Bioplotter, EnvisionTEC, Germany), pre extruded each group of bioink, and screened suitable groups for 3D-printing based on the fluidity of the bioink.

The porous scaffold was printed using a pneumatic extrusion-based 3D bioprinter, and a low-temperature printing platform was pre-cooled to −20 °C. The printing range was set to a diameter of 6 mm and a height of 2 mm using the built-in path planning software, with a 0/90° alternating printing path and a path spacing of 400 μm. The printing was performed at a pressure of 0.6 bar and a speed of 2 mm/s. After printing, the scaffold was transferred to a refrigerator and frozen for 2 days. By adjusting the freezing temperature, scaffolds with different nano-fiber surfaces were produced: PLA4 (4 °C), PLA-20 (−20 °C), PLA-80 (−80 °C), and PLA-196 (−196 °C). After freezing, the scaffolds were rinsed with deionized water. The washing procedure was conducted as follows: the scaffolds were rinsed with deionized water, with frequent water changes during the first 2 h. Subsequently, from 2 to 6 h, the water was replaced every 30 min. Finally, between 6 and 24 h, the water was refreshed approximately every 3 h.

The PLA-N scaffold was fabricated using 100 % PLA material extruded through a pneumatic extrusion-based 3D bioprinter (3D-Bioplotter, EnvisionTEC, Germany) at 210 °C. The printing parameters were configured through the built-in path planning software to create a 6 mm diameter, 2 mm height structure with a 0/90° alternating deposition path and 400 μm path spacing. The material cartridge was preheated to the target temperature in the printhead, followed by pre-extrusion calibration. Printing was subsequently performed at 0.6 bar pressure and 2 mm/s velocity. This melt-based fabrication process contrasts fundamentally with the solution-based phase separation approach used for other experimental groups, providing a baseline control for evaluating the specific contributions of the micro-nanofiber architecture generated by low-temperature phase separation.

### 2.2 Scanning electron microscopy

After washing, the scaffolds and material samples were metal-coated and observed using a scanning electron microscope (SEM) (S-3000N, Hitachi, Japan) at an accelerating voltage of 20 kV. The pore size and porosity were measured and quantified using ImageJ software (NIH, USA). If there were cells co-cultured on the scaffolds, the scaffolds were fixed with 4 % paraformaldehyde (PFA) for 24 h at 4 °C. Specimens were sequentially dehydrated in an ethanol gradient (70 %, 80 %, 90 %, 95 %, and 100 % v/v) and treated with hexamethyldisilazane (HDMS; Sigma-Aldrich, Canada) overnight to preserve structural integrity. Dehydrated scaffolds were sputter-coated with gold and imaged using SEM. Pseudocoloring of SEM micrographs was performed using Adobe Photoshop CC 2023 to enhance visualization. Cell spreading area and elongation ratios were quantified using ImageJ software (NIH, USA).

### 2.3 In vitro degradation study

The scaffolds were placed in sealed Petriplates containing simulated body fluid (SBF) and then incubated in vitro at 100 rpm and 37 °C. The SBF solution was prepared with the following ionic composition: NaCl (136.8 mM), NaHCO_3_ (4.2 mM), KCl (3.0 mM), K_2_HPO_4_ (1.0 mM), MgCl_2_·6H_2_O (1.5 mM), CaCl_2_ (2.5 mM), and Na_2_SO_4_ (0.5 mM). The pH of the solution was adjusted to 7.5 using tris (hydroxymethyl) aminomethane and hydrochloric acid. The SBF solution was replaced every 3 days to avoidany pH changes that may affect the degradation of the sample. After a predetermined time of in vitro degradation, each blendlm was washed with distilled water and then dried in vacuum. Finally, the weight loss of each sample was measured.

### 2.4 Compressive test

The 3D-printed PLA scaffolds were used as samples for compression testing. The dimensions and shape of the scaffolds were ensured to meet experimental standards. Compression tests were performed using a LLOYD universal testing machine (LR5K Plus, LLOYD, UK),with a compression rate of 1 mm/min, a maximum deformation limit of 80 %, and the use of cylindrical samples (6 mm diameter × 2 mm height). and the maximum stress (MPa), the corresponding stress-strain curves was determined.

### 2.5 Water contact angle (WCA)

The WCA on the scaffold surface was measured using a contact angle goniometer (PT-705A, Pusite Detection Equipment Co., Ltd., China). The sessile drop method was employed with a droplet analysis system (Theta Flex, Biolin Scientific, Sweden). Briefly, a 4 μL deionized water droplet was placed on the sample surface at room temperature and humidity, and images were captured after stabilization.

### 2.6 Atomic force microscopy (AFM)

The PLA scaffolds were placed on the AFM platform and secured using clamps and adhesives. The surface topography of each scaffold sample was scanned using an AFM, with three measurements taken at different locations.

### 2.7 Cell culture

In accordance with ethical guidelines for experimental animals, six 6-week-old male Sprague-Dawley (SD) rats were euthanized by cervical dislocation and sterilized with 75 % ethanol for 5 min. The tibia and femur of rats were aseptically removed, and the bone marrow was flushed to obtain a cell suspension. Red blood cells were lysed by incubation with a lysing buffer for 5 min, followed by centrifugation at 800 rpm. The cells were cultured in Dulbecco's Modified Eagle Medium (DMEM) (Gibco, UK) supplemented with 10 % fetal bovine serum (Gibco, UK) and 1 % penicillin-streptomycin (10,000 U/mL, Gibco, UK) for 24 h, after which the medium was replaced to obtain pure bone marrow stromal cells (BMSCs). The medium was refreshed every 2 days. After passaging the BMSCs to the third generation, the BMSCs were harvested for further experiments. All animal experiments were approved by the Laboratory Animal Ethics Committee of Jinan University. All experimental procedures were conducted in accordance with the Laboratory Animal Ethics Committee of Jinan University guidelines for the care and use of laboratory animals.

### 2.8 CCK-8 cell viability testing

Scaffolds were placed in 24-well plates, seeded with 1 × 10^5^ cells/mL BMSC cells in complete culture medium for 0, 24, 48, 72 h. At each time-point, 30 μL CCK-8 reagent (Dojindo) was added and incubated (37 °C, 5 % CO_2_, 2 h). The supernatant (100 μL) was transferred to a 96-well plate and absorbance was read at 450 nm. Background correction (medium only) was applied.

### 2.9 Co-culture of PLA scaffolds and BMSCs

PLA scaffolds were selected and sterilized by immersion in 70 % ethanol for 30 min, followed by thorough rinsing with sterile phosphate-buffered saline (PBS, 0.01 M, pH 7.4). The scaffolds were air-dried under a laminar flow hood to ensure sterility prior to cell seeding. BMSCs with a cell density of 1 × 10^5^ cells/mL were suspended in complete culture medium, then dropwise inoculated onto the surfaces of the PLA scaffolds to ensure uniform cellular distribution. The cell-seeded scaffolds were then transferred to six-well plates and submerged in complete medium. Cultures were maintained in a humidified incubator at 37 °C with 5 % CO_2_, and the medium was refreshed every 48 h.

### 2.10 Cytoskeletal staining

For cytoskeletal staining, samples were fixed with 4 % PFA (MultiSciences Biotech, China) at 24 h post-seeding, permeabilized with 0.1 % Triton X-100 (Solarbio, China) in PBS for 15 min, and washed three times with PBS. F-actin was stained with phalloidin probe (Abbkine, China) for 30 min in the dark, and nuclei were counterstained with 4′6-diamidino-2-phenylindole (DAPI; 10 μg/mL, Abbkine, China) for 30 s. Images were acquired using a confocal laser scanning microscope (LSM 880, Zeiss, Germany).

### 2.11 Alkaline phosphatase (ALP) activity assay

The osteogenic activity of the scaffolds was evaluated by measuring the ALP activity of BMSCs. DMEM supplemented with 10 % FBS, 1 mM dexamethasone (Solarbio, China), 50 μg/mL L-ascorbic acid 2-phosphate, and 10 mM β-glycerophosphate (Solarbio, China) was used as the osteogenic medium. After seeding 1 × 10^5^ BMSCs on the scaffolds, cell adhesion was ensured after 24 h. Cells were trypsinized after 7 days, and the supernatant was used to measure ALP activity using an ALP assay kit (Beyotime Biotechnology, China) at an absorbance of 450 nm.

### 2.12 Alizarin red staining

In order to evaluate the mineralized matrix, a total of 5000 BMSCs were seeded on each scaffold. After 24h, 1 mL of differentiation culture medium was added to each well, with subsequent medium changing every 3 days. On day 21 post-seeding, the scaffolds were washed with a 0.9 % NaCl solution, followed by fixation of the cells using 1 % glutaraldehyde. Following fixation, the calcium deposits were visualized by staining with a 2 % alizarin red solution for 45 min at room temperature.

### 2.13 DNA content assay

PLA scaffolds seeded with BMSCs were gently rinsed with PBS for 3 min to remove non-specific substances that might interfere with the assay. An appropriate amount of cell lysis buffer was added to each scaffold, and the scaffolds were gently shaken to ensure complete cell lysis and DNA release. The lysate was mixed with a dye according to the DNA assay kit instructions, and fluorescence intensity was measured using a microplate reader.

### 2.14 Quantitative real-time PCR (qRT-PCR)

Total RNA was extracted from cells or tissues using TRIzol reagent (Invitrogen, Carlsbad, USA) following the manufacturer's protocol. The extracted RNA was reverse-transcribed into cDNA, and real-time PCR was performed using the SYBR Premix Ex Taq II kit (Takara, China) on an ABI 7900 system (Applied Biosystems, USA). The expression levels of osteogenic-related and angiogenic-related genes, including *Runx2*, *Ocn*, *Opn*, *Col1a1, Macf1*, *HIF-1α*, *VEGF* and *α-SMA* were quantified.

Experiments were performed in triplicate and repeated 3 times. The primer sequences for each gene are listed in Table 2. Glyceraldehyde 3-phosphate dehydrogenase (*Gapdh*) was used as the endogenous control. Data were calculated as fold change relative to the BMSCs-CT group using the ΔΔCt method.

**Table 2:** Primer List for qRT PCR.

Gene	Forward Primer 5′ to 3′	Reverse Primer 5′ to 3′
Gapdh	AACTCCCATTCTTCCACCT	TTGTCATACCAGGAAATGAGC
Runx2	GAACCAAGAAGGCACAGAC	AATGCGCCCTAAATCACTG
Ocn	CTTTGTGTCCAAGCAGGAG	CTCCCAGCCATTGATACAG
Opn	TCTGACGAGGTCGTTGAGG	CTTTGCGATGCTGGTGCTC
Col-I	CAAGAAGACATCCCTGAAGTC	GCATACATCAGGTTTCCACG
Macf1	AGGACCTGGAGCTGATGGAG	TGGTGGTAGGTGGTGATGTT
HIF-1α	GACAGCCTCACCAAACAGAGC	TGGGACTATTAGGCTCAGGTG
VEGF	CTGCTGTCTTGGGTGCATTG	CACCGCCTTGGCTTGTCACA
α-SMA	GTCCCAGACATCAGGGAGTAA	TCGGATACTTCAGCGTCAGGA

### 2.15 Western blot

After 21 days of BMSCs cultured on the PLA scaffolds, cells were collected and lysed on ice using RIPA buffer (containing 1 mM PMSF; Solarbio) for 30 min. The lysate was centrifuged at 14,000 *g* for 20 min. Protein concentration was quantified using a BCA kit (Thermo Fisher Scientific, USA). Proteins were separated by polyacrylamide gel electrophoresis and transferred to a polyvinylidene fluoride (PVDF) membrane. The membrane was blocked with 5 % skim milk for 1 h and incubated with primary antibodies against Runx2 (1:500, Proteintech, China), Ocn (1:500, Abcam, UK), Opn (1:500, Proteintech, China), Col-I (1:1000, Proteintech, China), anti-Beta actin (1:20000, Proteintech, USA), and anti-Macf1 (1:1000, Abcam, UK) at 4 °C overnight. Horseradish peroxidase (HRP)-conjugated secondary antibodies (Pierce, Rockford, USA) were used, and protein bands were visualized using an ECL kit (Pioneer Biotechnology, China) and exposed to a chemiluminescence analyzer. The Image J software was utilized to analyze the grey values of protein expression for each group.

### 2.16 Transcriptome sequencing

Cells were collected from the scaffolds in each group. The cells were gently pipetted with TRIzol for 2 min, and the cell suspension was centrifuged. RNA was extracted using an RNA extraction kit, and high-throughput sequencing was performed on the Illumina HiSeq platform for data analysis.

Cells were collected from the scaffolds in each experimental group. The cells on the scaffolds were lysed by pipetting with TRIzol reagent for 2 min. A total of 250 μL of chloroform was added to 1 mL of the lysate, followed by vigorous shaking. The mixture was incubated at room temperature for 10 min and then centrifuged at 12,000×*g* for 5 min at 4 °C. The aqueous phase was transferred to a new microcentrifuge tube, mixed with 500 μL of isopropanol, and shaken thoroughly. After incubation at room temperature for 15 min, the RNA precipitate was washed with 1 mL of 75 % ethanol and centrifuged at 12,000×*g* for 10 min at 4 °C. The supernatant was discarded, and the RNA pellet was air-dried in a fume hood before being dissolved in 50 μL of DEPC-treated water. RNA concentration and purity were assessed using a Nanodrop spectrophotometer. The remaining RNA samples were stored at −80 °C for further analysis.

The purity and integrity of total RNA were evaluated using the Agilent 2100 BioAnalyzer system. Samples with an RNA Integrity Number (RIN) ≥ 8.0 were selected for downstream applications. mRNA was fragmented into short segments using a fragmentation reagent under moderate temperature in a thermomixer. The fragmented mRNA was used as a template for first-strand cDNA synthesis, followed by second-strand cDNA synthesis using a reaction system prepared according to the manufacturer's protocol. The double-stranded cDNA was purified, end-repaired, and adenylated at the 3′ end. Sequencing adapters were ligated, and cDNA fragments of the desired size were selected and amplified by PCR to construct the sequencing library. The quality of the constructed library was assessed using the Agilent 2100 Bioanalyzer and Qubit fluorometer. High-quality libraries were sequenced on an Illumina sequencing platform.

Raw sequencing data (raw reads) obtained from the Illumina HiSeq™ 2000 platform were subjected to quality control (QC) to ensure suitability for subsequent analyses. After QC, a series of bioinformatics analyses were performed, including gene and transcript quantification, principal component analysis (PCA), correlation analysis, condition-specific expression analysis, and differential gene expression screening. Additional analyses included exon quantification, gene structure optimization, alternative splicing detection, novel transcript prediction and annotation, SNP and Indel identification, and gene fusion detection.

Differentially expressed genes (DEGs) were further analyzed using Gene Ontology (GO) functional enrichment analysis, pathway enrichment analysis, cluster analysis, protein-protein interaction (PPI) network construction, and transcription factor prediction. These analyses provided deeper insights into the biological functions and regulatory mechanisms of the identified DEGs.

### 2.17 Cell transfection with plasmid

To knock down *Macf1* expression, *Macf1*-specific siRNA (Baili, China) was purchased. BMSCs were seeded into six-well plates and allowed to adhere for 24 h. For transfection, the cationic lipid reagent Lipofectamine 2000 (Thermo Fisher Scientific,USA) was used according to the manufacturer's protocol. BMSCs were transfected with 50 nM siRNA for 24 h. After transfection, cells were harvested for protein extraction, followed by Western blot analysis to evaluate *Macf1* expression levels.

To overexpress *Macf1*, the full-length coding sequence of the *Macf1* gene was first amplified by PCR. Following amplification, PCR products were purified and digested with restriction enzymes. The digested products were purified and ligated into a linearized pcDNA3.1 vector. After ligation, plasmids were transformed into *E. coli*, and bacteria were plated on agar plates containing ampicillin, followed by incubation at 37 °C for 15 h. Post-incubation, individual colonies were selected and screened via colony PCR to verify insert presence. Positive clones were sequenced to confirm insert sequence accuracy. For plasmid transfection, BMSCs were seeded into six-well plates at an appropriate density 24 h prior to transfection to ensure 90–95 % confluency at the time of transfection. Transfection was performed using 4 μg of pcDNA3.1-Macf1 plasmid DNA with Lipofectamine 2000 as the transfection reagent. After 6 h of incubation, the transfection medium was replaced with fresh complete medium containing serum. Cells were maintained at 37 °C in a 5 % CO_2_ atmosphere for 24 h. BMSCs were subsequently harvested for Western blot analysis to assess *Macf1* expression levels.

### 2.18 Establishment of rat calvarial critical-sized defect model

Based on pre-experimental results, 24 male SD rats aged 10 weeks were provided by the Zhuhai BesTest Bio-Tech Co.,Ltd. All rats were housed in a clean environment with access to food and water ad libitum, under controlled temperature (18–25 °C) and humidity (40–70 %) conditions, with a 12-h light/dark cycle (8:00–20:00). Bedding consisted of corn cobs and wood shavings, which were changed 2–3 times per week.

All experiments on animals were performed in accordance with the National Institutes of Health Guidelines for the Care and Use of Laboratory Animals, and approved by the Laboratory Animal Ethics Committee of Jinan University .10-week-old male SD rats were used as experimental animals. After anesthesia, a 6 mm diameter circular defect was created in the central region of the skull using a trephine, with a thickness of approximately 2 mm. The 2 mm thick, 6 mm diameter micro/nano-fiber scaffolds was co-cultured with BMSCs and induced for osteogenic differentiation before implantation.

The 24 rats were divided into 4 groups, with 6 rats in each group: Group A (CTRL): defect creation without scaffold implantation; Group B (PLA-N): implantation of a regular PLA scaffold; Group C (PLA4): implantation of a PLA4 biomimetic scaffold; Group D (PLA-196): implantation of a PLA-196 biomimetic scaffold. After 14 days of co-culture with BMSCs, the scaffolds were replaced with osteogenic induction medium for 7 days. The scaffolds were then implanted into the defects, ensuring contact with the dura mater, and the soft tissue and skin were sutured. Postoperatively, the rats were housed separately. At 4- and 8-weeks post-surgery, 3 rats from each group were euthanized by overdose anesthesia.

### 2.19 Micro-CT analysis of bone defects

To visually assess new bone formation at the injury site, scans were performed using a Scanco medical μCT40 (Scanco). Each sample (n = 3) was fixed by a foam plate, the scanning medium was air, the X-ray tube potential (peak) was 70 Kpv, the current was 270 μA, the integration time 300 ms, the voxel size was 18 μm^3^, and for each section, images were constructed by averaging 3 frames of acquisition. We scanned the entire skull sample, drew contour lines around the defect area and selected a 6 mm × 2 mm circular defect area as the volume of interest. Appropriate thresholds were selected to match the original grayscale images, 3D images were created from 2D slices using DICOM Viewer (RadiAnt). After 3D reconstruction, the trabecular thickness (Tb.Th) and bone volume fraction (BV/TV) of the new bone area were calculated using Image-Pro Plus version 5.0 (Media Cybernetics).

### 2.20 Histological analysis

After harvesting the calvarial bone samples, they were fixed with 4 % paraformaldehyde for 24 h. Decalcification was performed using 10 % ethylenediaminetetraacetic acid (EDTA, pH 7.2) (Leagene, China) at 4 °C. The samples were then dehydrated through a graded alcohol series, embedded in paraffin, and sectioned. Multiple sections were prepared from the center of the bone defect area, and the sections were subjected to routine hematoxylin and eosin (H&E) staining, as well as immunohistochemical staining for Opn (1:200, Proteintech, China), Ocn (1:200, Proteintech, China), and immunofluorescence staining for Macf1 (1:500, Proteintech, China), Col-I (1:500, Proteintech, China), Cd31 (1:500, Proteintech, China), and CD86 (1:200, Cell Signaling Technology, USA).

### 2.21 Tartrate-resistant acid phosphatase (TRAP) staining

Deparaffinized bone sections were incubated in 0.2 M acetate buffer (pH 5.0) containing 0.1 mg/ml naphthol AS-MX phosphate and 0.3 mg/ml fast red TR salt at 37 °C for 45 min, counterstained with Mayer's hematoxylin, and examined under light microscopy for TRAP-positive multinucleated osteoclasts.

### 2.22 Statistical analysis

Experimental data were processed using GraphPad Prism 8 software, and the results were presented as mean ± standard deviation. Student's t-test was used to compare differences between two groups, and one-way ANOVA was used to compare differences among multiple groups.

## 3 Results

### 3.1 Solvent ratio and printing parameter screening of PLA ink

**Fig. 1:**
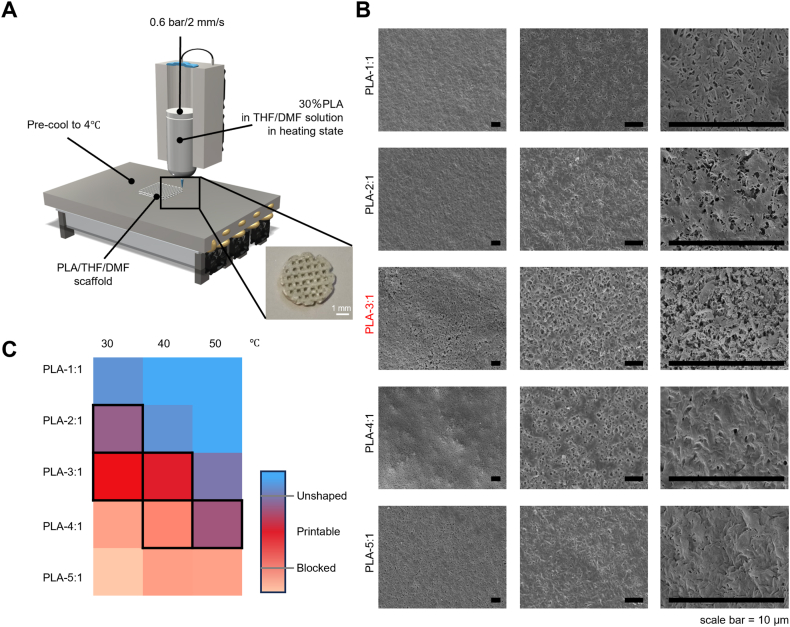
Using low-temperature thermally-induced phase separation (TIPS) technology and 3D printing to obtain porous structures. (A) Schematic diagram of the PLA scaffold preparation using a pneumatic extruder-based 3D bioprinter and TIPS. The inset shows a representative macroscopic image of the printed scaffold, scale bar: 1 mm. (B) SEM images of printed PLA, scale bar = 10 μm. (C) Heat map showing the stability of PLA scaffolds prepared at different solvent and temperatures.

We performed 3D printing using a pneumatic extrusion-based bioprinter equipped with a 400 μm diameter nozzle (Fig. 1A). The printed PLA scaffolds were cryogenically fixed in a freezer and subsequently subjected to aqueous washing-phase separation treatment. Notably, the rapid evaporation of THF solvent in the PLA bioink was found to cause frequent nozzle clogging, which has affected the extrusion of the PLA bioink. After parameter optimization, the washed scaffolds and material samples were analyzed via SEM. The PLA-3:1 group exhibited distinct nanoscale pores and micron-scale fibrous surface roughness, whereas the PLA-1:1 and PLA-2:1 group failed to develop such porous structures or textured surfaces. Although fibrous morphology was observed in the PLA-5:1 group, its microporosity remained sparse and poorly defined (Fig. 1B). According to the fluidity of extruded PLA bioink, we found that increasing the temperature from 30 °C to 50 °C will improve the fluidity of the material. The higher the proportion of DMF in the solvent, the stronger the fluidity of PLA bioink, but the worse the molding effect at the same time. On the other hand, the proportion of DMF was too small, and PLA bioink is easy to block the nozzle. We present the printability of PLA bioink in the form of heat map (Fig. 1C). The material group and temperature marked in black box are the parameters suitable for 3D printing. Optimal parameters were identified as a THF: DMF volumetric ratio of 3:1 combined with a printing temperature of 40 °C.

### 3.2 Temperature of thermally-induced phase separation affects the forming effect of scaffold fiber surface

**Fig. 2:**
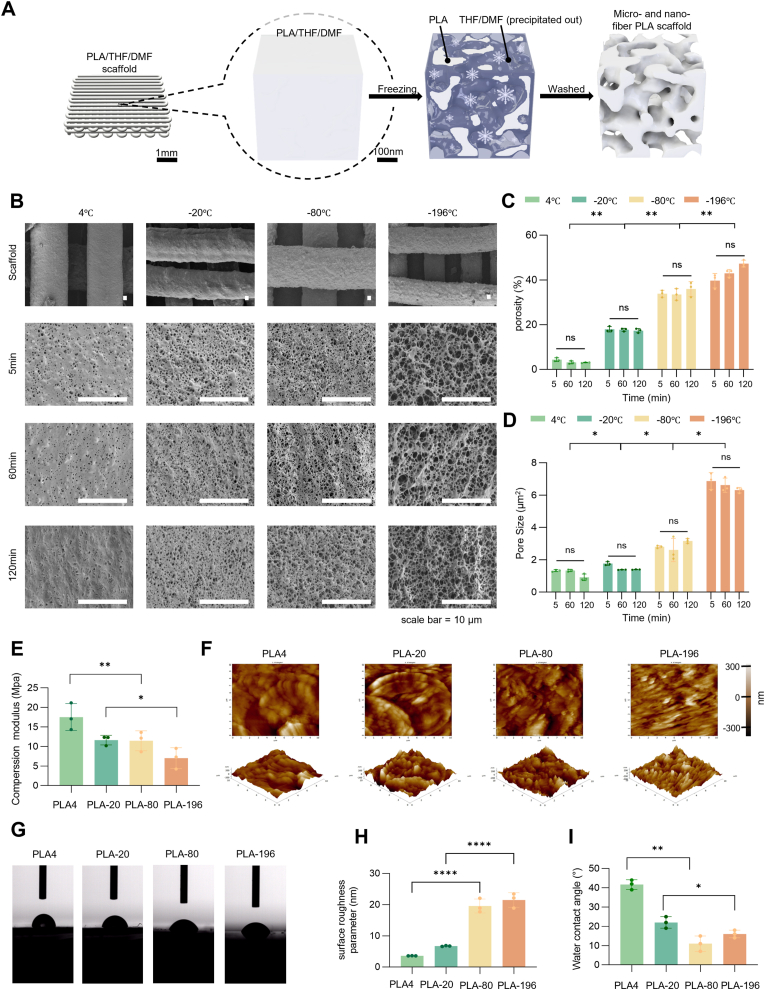
Preparation and characterization of PLA scaffolds with different micro- and nano-structures. (A) Schematic diagram showing that the solvent is removed by a freezing water-washing frame, achieving a micro- and nano-fiber structure guided by the TIPS process. (B) SEM images of PLA scaffolds under different treatments of time and temperature. (C) Quantitative analysis of porosity in PLA scaffolds under different treatments. (D) Quantitative analysis of pore size in PLA scaffolds under different treatments. (E) Quantitative analysis of compressive modulus (MPa) of PLA scaffolds under different treatments. (F) AFM images of PLA scaffolds under different treatments. (G) Water contact angle images of PLA scaffolds under different treatments. (H) Surface roughness quantification based on AFM (n = 3). (I) Water contact angle measurements of PLA scaffolds under different treatments. *: *p* < 0.05, **: *p* < 0.01, ****: *p* < 0.0001, ns: *p* > 0.05.

We used PLA-3:1 bioink for 3D printing the grid-shaped scaffolds. Scaffolds were printed on a −20 °C plane and were formed effectively. After printing, the scaffolds were immediately placed in a frozen environment at different temperatures for TIPS, after 5–120 min, scaffolds were then washed with deionized water pre cooled at 4 °C (Fig. 2A). SEM analysis revealed that the produced scaffolds displayed consistent pore wall thickness and size (Fig. 2B). A decrease in the TIPS temperature led to a gradual increase in the porosity of the scaffold surface's porous structure, showing significant differences between adjacent temperatures (*p* < 0.01). Additionally, the pore size of the porous structure increased progressively. Specifically, when subjected to liquid nitrogen (−196 °C), the pore size of the micro- and nano- fibrous porous structure on the scaffold surface reached 6.01 ± 2.06 μm^2^, the largest among all groups. Subsequent freeze-washing at 4 °C, −20 °C, and −80 °C, resulted in pore sizes of 1.158 ± 0.24 μm^2^, 1.33 ± 0.77 μm^2^ and 2.75 ± 1.23 μm^2^, respectively (Fig. 2C). Notably, the duration of freezing did not significantly affect the porous structure of the scaffold surface (*p* > 0.05) (Fig. 2D). Therefore, following the printing process with PLA-3:1, a 5-min freeze-washing of the scaffold can generate a micro- and nano-fibrous structure on the surface. Next, in order to investigate the characterization of the scaffolds at different TIPS temperatures, the scaffolds are named according to the temperature used for each group, such as PLA4, PLA-20, PLA-80, and PLA-196.

The compression test results clearly indicated that the scaffold demonstrated a significantly higher compressive modulus of 17.93 ± 0.27 MPa when undergoing TIPS at 4 °C, in comparison to other groups. The group with the lowest compressive modulus recorded was 9.83 ± 5.12 MPa (Fig. 2E) Furthermore, we have characterized their mechanical performance through stress-strain curves (Fig. S1). The surface topography of each group of scaffolds were examined using AFM. Quantitative analysis of the surface roughness parameter (Sq) revealed that the average surface roughness at 4 °C was 3.62 ± 0.04 nm, significantly lower than the other groups. Conversely, scaffolds processed at −80 °C and −196 °C displayed significantly higher values of 19.90 ± 2.17 nm and 20.16 ± 3.27 nm, respectively (Fig. 2F–H). This trend indicates a clear correlation between the phase-separation temperature and the resulting surface topography, with lower processing temperatures leading to substantially increased surface roughness. Water contact angle measurements were conducted to assess the hydrophilicity of the scaffolds. The results indicated that scaffolds processed at 4 °C exhibited the highest contact angle of approximately 41.26 ± 4.78 nm, while those processed at −196 °C demonstrated superior hydrophilic properties (Fig. 2G–I). Furthermore, CCK-8 assays conducted across the four scaffold variants revealed ubiquitous cell proliferation, with PLA-196 scaffolds demonstrating significantly enhanced cellular expansion compared to all other groups (Fig. S2).

### 3.3 The micro- and nano-fiber structure affects the early adhesion behavior of BMSCs on the PLA scaffold

**Fig. 3:**
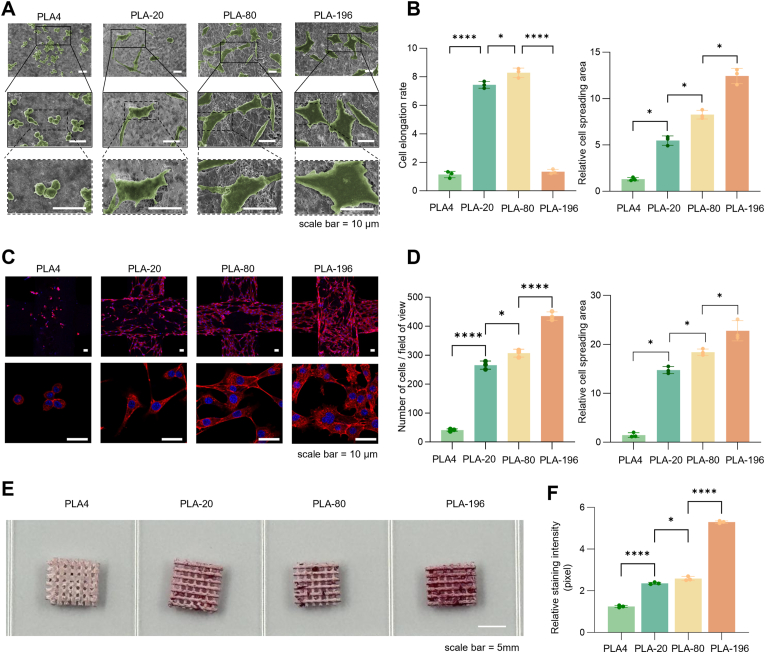
Early adhesion behavior of BMSCs on the scaffold surface at 24 h. (A) SEM images of BMSCs cultured on PLA scaffolds, scale bar = 10 μm. (B) Quantitative analysis of cell elongation ratios and relative spreading areas. (C) Fluorescence images of BMSCs cultured on PLA scaffolds, red = cytoskeleton stained with phalloidin probe, blue = nucleus stained with DAPI, scale bar = 10 μm. (D) Cell count and relative spreading areas of BMSCs on PLA scaffolds. (E) Alizarin Red S staining of BMSCs cultured on PLA scaffolds. (F) Quantitative statistics of Alizarin Red S staining. ns: *p* > 0.05, *: *p* < 0.05, ****: *p* < 0.0001. (For interpretation of the references to color in this figure legend, the reader is referred to the Web version of this article.)

After adding cell suspension droplets onto each group of PLA scaffolds for 24 h, the SEM images indicated significant morphological distinctions in BMSCs when cultured on different micro- and nano- fiber surfaces (Fig. 3A). Cells in the PLA4 group, predominantly displayed a spherical morphology with short filopodia at the periphery. Conversely, cells in the PLA-20 and PLA-80 groups exhibited a spread morphology contrasting significantly with the spherical shape of the PLA4 group and the polygonal morphology seen in the PLA-196 group. Notably, cell proliferation densities also exhibited marked variations. Elongation analysis revealed that PLA-80 and PLA-20 groups possessed the highest cellular elongation ratios, while PLA-196 cells showed values closer to 1. From PLA4 to PLA-196, as the surface porosity of the nanofibers increased and pore size expanded, the PLA-196 group exhibited significantly greater cell spreading than the other three groups (Fig. 3B).

We also observed differences in cell growth morphology and density on different scaffolds through cytoskeleton staining (Fig. 3C), BMSCs were spherical on PLA4, but fully spread and grew on PLA196. Specifically, PLA-196 demonstrated both higher cell density (435.56 ± 13.25 cells/mm^2^, representing a 10-fold increase compared to PLA4) and broader spreading extent, with cells positioned in closer proximity. The cell spreading areas across all four groups followed trends consistent with our SEM observations. These findings clearly indicate that enhanced surface porosity and pore dimensions in scaffolds correlate with improved BMSC proliferation capacity and more extensive spreading (Fig. 3D). The fluorescence intensity exhibited a consistent trend. (Fig. S3). To functionally assess the terminal stage of osteogenic differentiation, we evaluated the extracellular matrix mineralization of BMSCs cultured on the various PLA scaffolds after 21 days in osteogenic induction medium using Alizarin Red S staining. As shown in Fig. 3 E and F, the staining intensity followed a clear gradient, with the PLA-196 scaffold exhibiting the most intense red coloration, indicative of the highest level of calcium accumulation. The PLA-4 scaffold showed the weakest staining, while the PLA-20 and PLA-80 groups displayed intermediate and progressively increasing levels of mineralization, respectively.

### 3.4 mRNA sequencing reveals that *Macf1* regulates the cell morphology and osteogenic differentiation of BMSCs on the scaffold

**Fig. 4:**
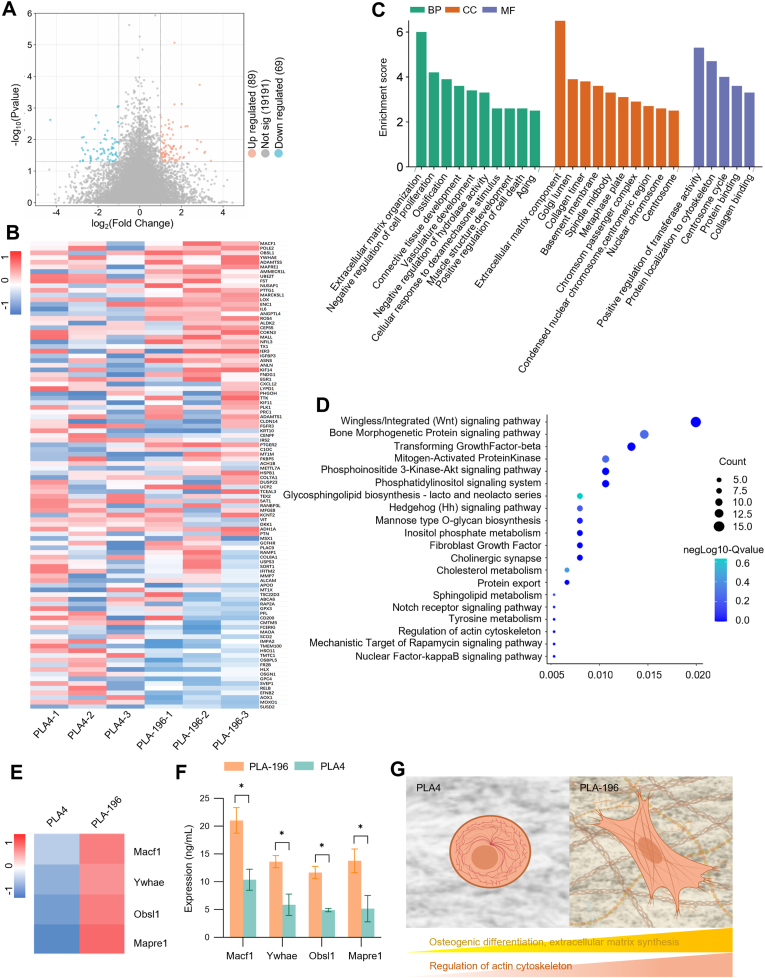
mRNA sequencing analysis of BMSCs cultured on TIPS scaffolds. BMSCs were cultured on PLA4 and PLA-196 scaffolds for 7d, (A) Volcano plot of DEGs. (B) GO analysis of 2-fold DEGs. BP-BiologicalProcess, CC-Cellular Component, BF-Biological Function. (C) Heatmap of 2-fold DEGs. (D) KEGG analysis of 2-fold DEGs. (E) Heatmap of expression levels of cytoskeletal related genes in PLA4 and PLA-196 groups among 2-fold DEGs. (F) ELISA detection of expression levels of cytoskeletal related proteins in PLA4 and PLA-196 groups. (G) A schematic illustrating the effect of cellular state on related pathways. *: *p* < 0.05.

Gene microarray analysis further elucidated the differences in mRNA expression of BMSCs cultured on micro- and nano-fiber surfaces with varying porosity and pore size. Specifically, we compared the PLA4 and PLA-196 groups, which exhibited the greatest disparity in cell spreading. Volcano plot analysis of the transcriptomic data revealed differentially expressed genes (DEGs) between these two groups. This analysis identified 69 down-regulated genes (blue points), 89 up-regulated genes (orange points), and 19,191 genes without significant changes (grey points) (Fig. 4A). A heatmap visualization provided an intuitive representation of the expression patterns. This revealed that genes promoting osteogenic differentiation, cell migration, and proliferation generally exhibited higher expression levels in the PLA-196 group compared to the PLA4 group. Notably, the PLA-196 group showed elevated expression of genes associated with cell proliferation and bone formation, such as *Runx2*, *Alpl*, and *Bmp2* (Fig. 4B).

To investigate the biological processes associated with these DEGs, Gene Ontology (GO) enrichment analysis was performed. Significantly enriched biological processes among the DEGs in the PLA4 versus PLA-196 comparison included those related to cell proliferation, migration, differentiation, and osteogenic differentiation (Fig. 4C). Specifically, pathways associated with the regulation of cell morphology were highly enriched within the Biological Process (BP) and Cellular Component (CC) categories, achieving an enrichment score of 6. Enrichment was also evident for pathways related to cell proliferation and vascular endothelial development within the BP category (Fig. 4C). Furthermore, KEGG pathway enrichment analysis of the DEGs identified enrichment of osteogenesis-related signaling pathways, including the Wnt signaling pathway and the PI3K-Akt signaling pathway, in both groups. This further supports the importance of these pathways in the osteogenic differentiation of BMSCs (Fig. 4D). Building upon the pathway analysis, we sought to identify pivotal upstream regulators that could functionally link the nanotopographical sensing to the activation of these pro-osteogenic pathways. The transcriptomic data pointed to a compelling candidate: Microtubule-Actin Crosslinking Factor 1 (Macf1). Macf1 is a large cytoskeletal scaffold protein known to be critical for cell shape determination by integrating microtubules and actin filaments. Importantly, emerging evidence positions Macf1 as a key modulator and effector of the Wnt/β-catenin signaling pathway, where it facilitates the cytoplasmic stabilization of β-catenin. The concurrent enrichment of “regulation of cell morphology” in our GO analysis and the Wnt signaling pathway in our KEGG analysis provides a rationale to hypothesize that Macf1 serves as a central node, transducing the biomechanical cues from the PLA-196 scaffold's architecture into a biochemical signal that drives osteogenic specification.

To validate whether the micro- and nano-fiber surface promotes osteogenic differentiation by modulating cell morphology, the expression levels of key candidate genes (*Macf1*, *Ywhae*, *Obsl1*, *Mapre1*) were assessed via ELISA. The results demonstrated significantly elevated expression levels of these genes in the PLA-196 group, with the most pronounced upregulation observed for Macf1 (21.03 ± 2.29 ng/mL) compared to the PLA4 group (10.37 ± 1.90 ng/mL) (Fig. 4E and F). Given the differences in cell adhesion and growth behavior observed on scaffolds treated at different TIPS temperatures in our previous studies, and in conjunction with the results of transcriptome sequencing, we preliminarily hypothesize that the differential expression of the Macf1 molecule may closely affect the cell morphology of BMSCs (Fig. 4G). Moreover, the differences in osteogenic differentiation levels suggested by the transcriptome results will also become an aspect of our subsequent research.

### 3.5 The PLA-196 scaffolds with larger pore size and higher porosity enhances osteogenic in BMSCs

**Fig. 5:**
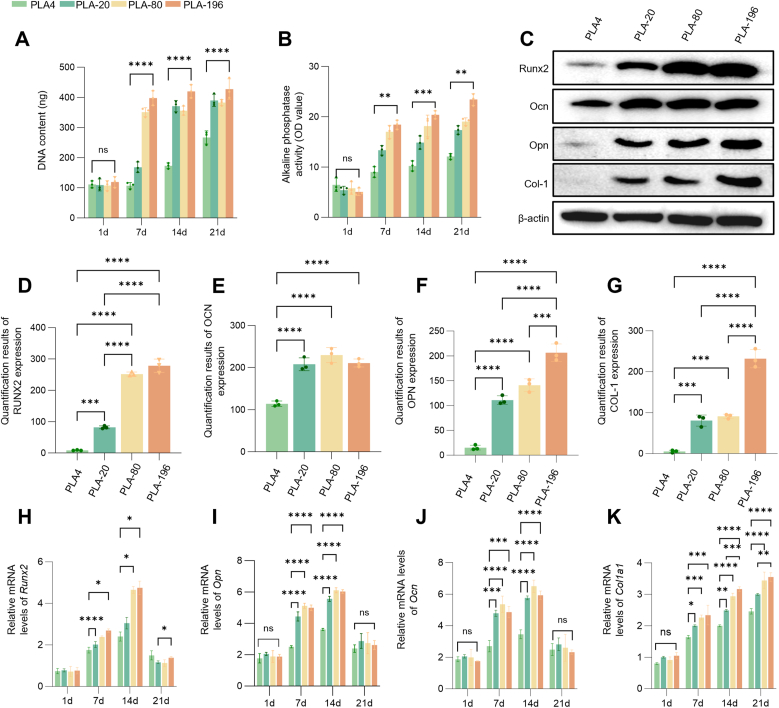
Osteogenic differentiation of BMSCs on the scaffolds over 21 days. (A) DNA quantification reflecting BMSC proliferation up to day 21. (B) Temporal ALP activity indicating osteogenic commitment. (C, D) Western blots and quantification of osteogenesis-related proteins (Runx2, Ocn, Opn, Col-1) at day 7. (E–K) qRT-PCR of *Runx2*, *Ocn*, *Col1a1* and *Opn* mRNA levels across 21 days. ns: p > 0.05, *: p < 0.05, **: p < 0.01, ***: p < 0.001, ****: p < 0.0001.

The number of BMSCs on different scaffolds was assessed using a DNA quantification kit. The PLA-196 group reached a high level by day 7, with no significant increase at 14 or 21 days. In contrast, the PLA-80 and PLA-20 groups plateaued at day 14, while the PLA4 group showed a continuous rise in DNA content throughout 21 days (Fig. 5A). The stabilization of DNA content indicates that BMSC proliferation on the scaffolds had ceased, likely due to lack of available growth space. Since all scaffolds share a macroporous grid structure, the similar maximal DNA levels across groups suggest that the fiber surface produced by TIPS processing does not appreciably enlarge the available space for cell growth. As a marker of osteogenic differentiation, ALP activity continued to rise throughout the 21-day period. The PLA-196 group demonstrated significantly enhanced ALP activity, increasing from 5.07 ± 0.74 U/L to 23.43 ± 1.16 U/L. Moderate activity was observed in PLA-80 scaffolds, while PLA-20 showed further reduction, and PLA4 exhibited minimal expression (Fig. 5B). To functionally assess the terminal stage of osteogenic differentiation, we evaluated the extracellular matrix mineralization of BMSCs cultured on the various PLA scaffolds after 21 days in osteogenic induction medium using Alizarin Red S staining. As shown in Fig. 3 E,F, the staining intensity followed a clear gradient, with the PLA-196 scaffold exhibiting the most intense red coloration, indicative of the highest level of calcium accumulation. The PLA-4 scaffold showed the weakest staining, while the PLA-20 and PLA-80 groups displayed intermediate and progressively increasing levels of mineralization, respectively.

Expression of osteogenesis-related proteins Runx2, Ocn, Opn, Col-1 in BMSCs was quantified by Western blotting at day 7 (Fig. 5C and D). Runx2 expression progressively increased across PLA scaffolds, with significantly elevated levels in PLA-80 and PLA-196 groups (∼3-fold increase vs. PLA-20). PLA-4 maintained relatively low expression. Ocn expression was low in PLA-4. While comparable among PLA-20, PLA-80, and PLA-196 groups, the strongest expression occurred in PLA-196. Opn and Col-1 followed similar expression patterns: lowest in PLA-4, comparable between PLA-20 and PLA-80, and highest in PLA-196. PLA-196 scaffolds consistently demonstrated superior Col-1 expression across all porosity variants. mRNA levels of osteogenic differentiation markers *Opn* exhibited a dynamic biphasic pattern across all scaffolds: expression increased initially, peaked at day 14 (3-fold vs. day 1), and subsequently declined. In contrast*, Runx2, Ocn, Col1a1* mRNA levels showed a sustained increase throughout the culture period (Fig. 5E–K). The above findings indicate that PLA-196, characterized by its larger pore size and higher porosity, exhibits a significantly more pronounced capacity to promote osteogenic differentiation of BMSCs.

### 3.6 *Macf1* expression positively correlated with the cell spreading and osteogenic differentiation of BMSCs

**Fig. 6:**
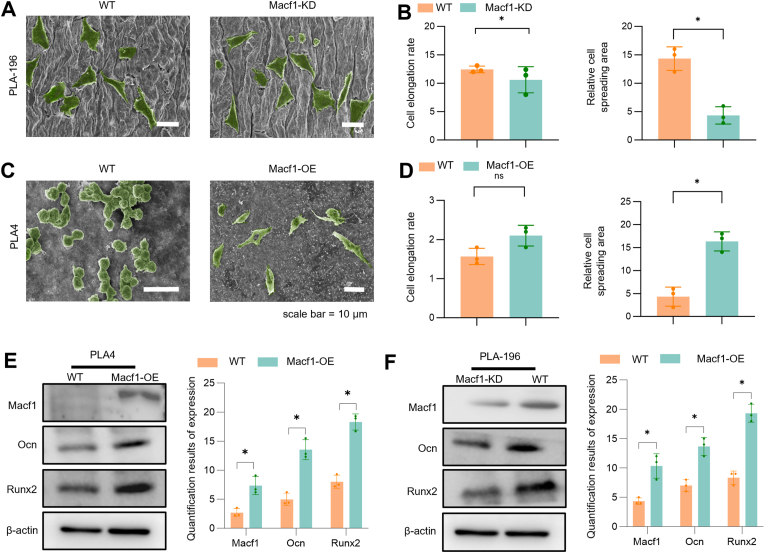
Macf1 promotes osteogenesis by modulating the cytoskeleton. (A) SEM images of WT and Macf1-KD BMSCs seeded on PLA-196 scaffolds, scale bar = 10 μm. (B) Elongation rate and spreading area of WT and Macf1-OE BMSCs. (C) SEM images of WT and Macf1-OE BMSCs seeded on PLA4 scaffolds, scale bar = 10 μm. (D) Elongation rate and spreading area of WT and Macf1-OE BMSCs. (E) Expression levels of post-Macf1-OE osteogenic marker proteins on PLA4 scaffolds. (F) Expression levels of osteogenic marker proteins following MACF1 on PLA-196 scaffolds. ns: *p* > 0.05,*: *p* < 0.05.

To investigate whether *Macf1* promotes osteogenesis through cytoskeletal regulation, BMSCs with *Macf1* knockdown (Macf1-KD) or overexpression (Macf1-OE) were constructed and seeded onto PLA-196 and PLA4 scaffolds, respectively (Fig. 6A–C; green pseudo-color indicates cells). On PLA-196 scaffolds, WT cells exhibited well-spread morphology with tight intercellular connections, defined edges, and well-developed pseudopodia, indicative of a mature phenotype. The average spreading area was approximately 14.07 ± 2.28 μm^2^, with an elongation rate of approximately 3.63 ± 0.84. In contrast, Macf1-KD cells displayed significantly reduced spreading area decreased to 4.52 ± 2.11 μm^2^ and a markedly increased elongation rate, suggesting a contracted morphology (Fig. 6B). On PLA4 scaffolds, WT cells appeared rounded with minimal spreading (3.54 ± 2.06 μm^2^), while Macf1-OE cells exhibited an approximately 4.5-fold increase in spreading area. The elongation rate also increased when *Macf1* overepressed (Fig. 6D).

Further analysis of osteogenic protein expression revealed that on PLA4 scaffolds, Macf1-OE cells exhibited significantly elevated levels of Macf1, Runx2, and Ocn compared to WT controls (Fig. 6E). Macf1 expression increased approximately threefold, Runx2 levels rose from 5.02 ± 1.71 to 13.59 ± 2.16, and Ocn expression increased from 8.00 ± 1.44 to 17.50 ± 2.35, indicating that Macf1 upregulation enhances osteogenic protein expression. Conversely, on PLA-196 scaffolds, Runx2 and Ocn expression was significantly downregulated in Macf1-KD cells (Fig. 6F). Collectively, these results demonstrate that *Macf1* plays a critical role in promoting cell adhesion, cell growth and osteogenic differentiation.

### 3.7 The PLA scaffolds repaired skull defects in SD rats

**Fig. 7:**
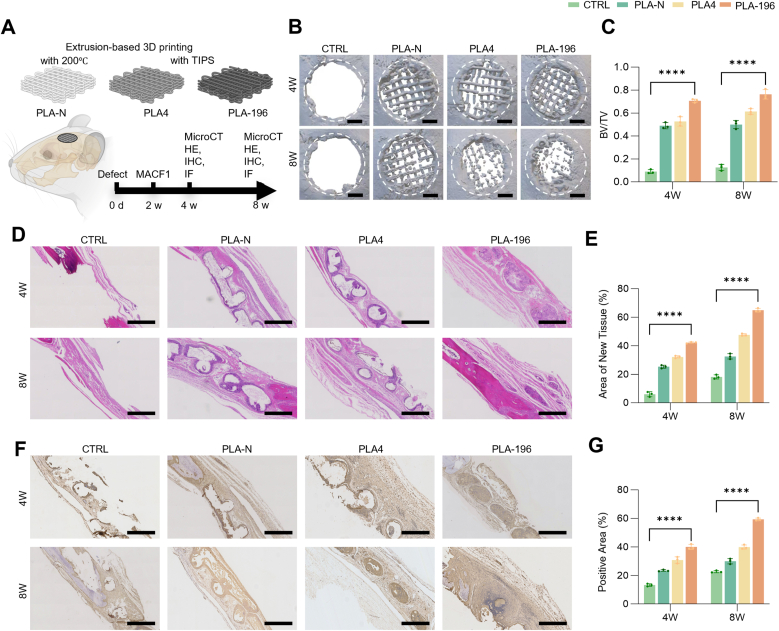
PLA scaffolds enhance bone regeneration in SD rat cranial bone defect models. (A) Schematic diagram of the SD rat cranial bone defect models. The CTRL group received no treatment, while the PLA-N group had PLA-N scaffolds implanted, the PLA4 group had PLA4 scaffolds implanted, and the PLA-196 group had PLA-196 scaffolds implanted. The defect size was 6 mm. (B) Micro-CT images of cranial bone defects at 4 and 8 weeks, n = 3, scale bar = 1 mm. (C) Bone volume fraction (BV/TV) scores. (D) HE staining of new bone tissue in the defect areas at 4 and 8 weeks, scale bar = 500 μm. (E) Quantification of the proportion of new bone tissue area. (F) Ocn immunohistochemical staining of new bone tissue in the defect areas at 4 and 8 weeks, scale bar = 500 μm. (G) Ocn-positive area, scale bar = 500 μm ****: *p* < 0.0001.

As shown in Fig. 7 A, three kinds of PLA scaffolds were implanted into the cranial bone defects of SD rats, among them, PLA4 and PLA-196, as mentioned earlier, PLA-N scaffold was prepared using high-temperature 3D printing. In theory, PLA-N does not have a micro- and nano fiber surface structure. Micro-CT analysis revealed new bone formation across all groups at 4- and 8-weeks post-operation (Fig. 7B). However, the PLA-N and PLA4 groups exhibited sparse bone deposition around scaffolds with incomplete degradation at 8 weeks. We also performed in vitro degradation testing on the scaffolds (Fig. S6). In contrast, the PLA-196 group exhibited accelerated degradation (at 8 weeks) and significantly enhanced osteogenesis, achieving 62.13 ± 3.52 % bone volume fraction (BV/TV) at 8 weeks, 1.5-fold higher than PLA-N and PLA4 (Fig. 7C). The quantitative microstructural analysis further revealed a significant increase in trabecular number (Tb.N) accompanied by a concurrent decrease in trabecular separation (Tb.Sp) across the scaffords (Fig. S4).

Our analysis of the PLA-196 group revealed unparticularly significant. changes in micro-CT parameters between the 4- and 8-week time points. We attribute this observation to a crucial biological phenomenon-the “masking effect” of new tissue ingrowth—wherein rapid bone formation within the scaffold pores progressively replaces the degrading polymer while maintaining the overall structural architecture. This biological substitution process thereby maintains radiographic continuity even as material degradation proceeds.

H&E staining (Fig. 7D and E) demonstrated that minimal new bone in CTRL group (<15 % area), with progressive fibrosis at 8 weeks.; however, the regeneration bone tissue remained relatively low. PLA-N scaffolds retained residual material at 4 weeks and sparse new bone formation was observed specifically at the implant-tissue interface. By 8 weeks, sparse new bone trabeculae bone had formed around the scaffold. PLA4 scaffolds induced active osteoblast recruitment at 4 weeks, achieving 42.01 ± 2.71 % bone coverage by 8 weeks. Remarkably, PLA-196 scaffolds exhibited rapid degradation and dense trabecular bone formation, occupying >60 % of defect areas at 8 weeks. Critically, the bone tissue induced by the PLA-196 scaffold exhibited significantly greater density and maturity at both time points compared to the other groups.

Immunohistochemical staining for Ocn further confirmed the aforementioned findings, with the positive areas corresponding to the regions of new bone growth observed in the HE staining results. Additionally, quantitative analysis of the positive areas revealed that the Ocn-positive areas in PLA-196 group reached 40.65 ± 1.84 %, significantly higher compared to the other three groups (Fig. 7F, G,S5). TRAP staining for osteoclasts was uniformly negative across all groups; no multinucleated, giant-cell-like TRAP-positive osteoclasts were observed within the bone tissue. These findings indicate that the healing process is still in the bone-regeneration phase and has not yet progressed to the remodeling stage (Fig. S7).

### 3.8 Influence of PLA scaffolds on proteins involved in repaired skull in SD rats

**Fig. 8:**
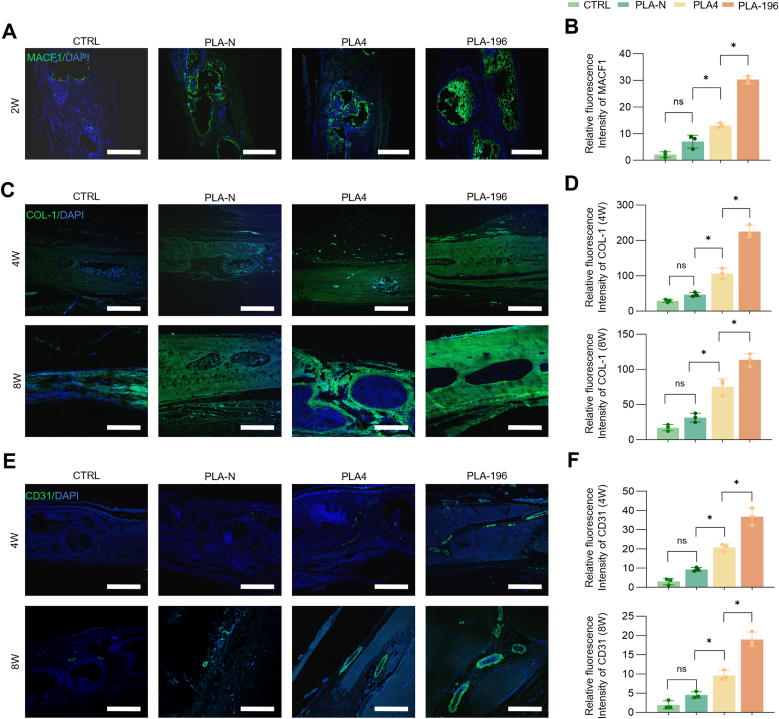
Spatiotemporal expression profiles of osteogenic markers in SD rat calvarial defect models. (A) Immunofluorescence staining of Macf1 at 2 weeks post-implantation, scale bar = 200 μm. (B) Quantification of Macf1 relative fluorescence intensity. (C) Immunofluorescence staining of Col-I at 4 and 8 weeks, scale bar = 200 μm. (D) Quantification of Col-1 relative fluorescence intensity in. (E) Immunofluorescence staining of CD31 expression (neovascular marker) at 4 and 8 weeks, scale bar = 200 μm. (F) Quantification of CD31 relative fluorescence intensity. ns: *p* > 0.05, *: *p* < 0.05.

To further verify the expression levels of key proteins involved in osteogenic repair in vivo, immunofluorescence staining was performed. Immunofluorescence staining confirmed spatiotemporal enrichment of Macf1 during bone regeneration (Fig. 8A). At 2 weeks post-implantation, PLA-196 scaffolds induced substantial Macf1 accumulation at scaffold-bone interfaces, whereas minimal enrichment was observed in the CTRL group (Fig. 8B). PLA-N and PLA4 showed intermediate Macf1 expression, with fluorescence intensity shows scaffold-dependent gradient change (Fig. 8A and B). Col-I distribution revealed progressive matrix maturation (Fig. 8C). By 4 weeks, PLA-196 exhibited dense, continuous Col-I networks permeating defect areas, contrasting with sparse, fragmented fibers in CTRL (p < 0.001) and PLA-N. PLA4 displayed focal Col-I deposition around nuclei but lacked structural continuity (Fig. 8C and D). Neovascularization were assessed via CD31 staining (Fig. 8E and F). At 4 weeks, PLA-196 demonstrated interconnected CD31 tubular structures (32.7 ± 2.8 % area), whereas CTRL and PLA-N showed only scattered signals. PLA4 formed isolated CD31 tubules but failed to establish functional networks. By 8 weeks, PLA-196 developed mature, branched vasculature with vessel density increasing 2.1-fold versus PLA4 (Fig. 8F). Immunofluorescence staining for COL-1 and CD86 revealed no substantial M1 macrophage infiltration in the newly formed bone tissue across all groups, indicating that PLA scaffold degradation did not elicit a local inflammatory response (Fig. S8). Consistent with the immunofluorescence findings, gene expression analysis of key angiogenesis markers further substantiated the superior pro-angiogenic capacity of the PLA-196 scaffold. qRT-PCR results demonstrated that the PLA-196 group exhibited significantly elevated mRNA levels of HIF-1α and VEGF at both 4 and 8 weeks compared to the CTRL, PLA-N, and PLA4 groups (Fig. S9). In contrast, the expression of α-SMA, a marker associated with mature vasculature, showed no significant differences among the groups at either time point.

## 4 Discussion

The advancement of hierarchical scaffolds featuring integrated macroporous frameworks and micro-nano fibrous surfaces represents a transformative approach in bone regeneration [[29], [30], [31], [32]]. These dual-scale architectures operate synergistically: the macroporous networks (400 μm spacing in our design) facilitate essential biological processes including vascular infiltration, nutrient diffusion, and cellular migration, while the nanofibrous topographies (100–300 nm diameter) replicate the structural and mechanical properties of native extracellular matrix [[33], [34], [35], [36]]. This biomimetic interface significantly enhances adsorption of key adhesive proteins such as fibronectin and vitronectin, creating optimal conditions for integrin-mediated cellular adhesion [37]. The porous scaffold was printed using a pneumatic extrusion-based 3D bioprinter, and a low-temperature printing platform was pre-cooled to −20 °C. This precisely controlled thermal environment enables rapid thermodynamic quenching upon deposition, inducing instantaneous liquid-liquid phase separation that preserves biphasic nanostructures before polymer relaxation, thereby ensuring reproducible fabrication of 3D scaffolds with high porosity, optimal interconnectivity, and structural fidelity essential for bone tissue engineering.

We posit that temperature serves as a critical factor in the formation of micro-nanofiber surfaces during low-temperature phase separation processing. It directly influences the phase separation kinetics and crystallization degree within the polymer solution, thereby determining the final morphology, pore size, and porosity of the resulting micro-nanofibers. This precisely controlled thermal environment enables rapid thermodynamic quenching upon deposition, inducing instantaneous liquid-liquid phase separation that preserves biphasic nanostructures before polymer relaxation, thereby ensuring reproducible fabrication of 3D scaffolds with high porosity, optimal interconnectivity, and structural fidelity essential for bone tissue engineering. Theoretically, even lower cryogenic media (e.g., liquid helium, −269 °C) could further increase porosity. However, such extreme cooling would induce more severe and rapid thermodynamic instability, it could also compromise the scaffold's ability to meet the basic mechanical requirements for implantation. Based on degradation assessments of various scaffolds both in vitro and in vivo, we conclude that higher porosity promotes scaffold degradation by enhancing its accessibility to endogenous enzymatic agents in physiological environments. The scaffold also exhibited a degradation trend in vitro, albeit less pronounced than observed in vivo, which may be attributed to the absence of enzymatic and cellular reactions present in the physiological environment that could not be effectively replicated in the simulated conditions.

The profound biological impact of this architectural strategy is evidenced by the tenfold increase in bone marrow stromal cell density observed on our micro-nano fibrous PLA-196 scaffolds compared to conventional surfaces, alongside accelerated osteogenic differentiation confirmed through alkaline phosphatase activity and osteocalcin expression. These findings align with and extend previous work on strontium-modified beta-tricalcium phosphate scaffolds, where similar topological features elevated Runx2 and vascular endothelial growth factor expression, substantiating the critical role of hierarchical design in coordinating vascularized bone regeneration [31,38].

The fabrication of such complex architectures has historically presented substantial technological challenges. Conventional techniques including freeze-drying and particle leaching offer limited capacity for precise pore geometry control, often resulting in structural inconsistencies that compromise functionality. Electrospinning, while capable of generating high-quality nanofibers, typically produces structures with insufficient thickness for clinical implementation, restricting cellular infiltration to superficial layers. Polymer-induced phase separation methods can achieve nanofibrous structures but frequently depend on solvents with inherent biocompatibility concerns, such as hexafluoroisopropanol, or require cytotoxic crosslinkers like genipin [39]. The integration of additive manufacturing with phase separation physics represents a significant methodological evolution. Particularly relevant is the pioneering work by Ana Colette Maurício employing photopolymerization-induced phase separation integrated with digital light processing printing. Their innovative approach leveraged thermodynamic incompatibility between polyethylene glycol diacrylate and hydroxyapatite nanoparticles during ultraviolet curing, successfully generating sub-200 nm fibrous architectures within precisely defined macrogeometries [40]. Crucially, these scaffolds demonstrated a 3.2-fold enhancement in tensile strength attributable to nanoscale interfacial reinforcement, though the acrylate-based resin system necessitated photoinitiators that reduced bone marrow stromal cell viability by 63 % at day seven, highlighting persistent biocompatibility limitations in photochemical approaches [41]. Kongchang Wei's research similarly employed phase separation technology, utilizing stannous salts as catalysts and macromolecular materials as fusion agents to prepare PLA scaffolds under high-temperature conditions. However, this process is complex and exhibits low raw material utilization [42].

Our thermally driven methodology fundamentally addresses these constraints through the strategic selection of polylactic acid, a clinically established polymer requiring no reactive monomers or photoinitiators. Central to this process is the sophisticated engineering of the solvent system. The tetrahydrofuran and N, N-dimethylformamide co-solvent ratio was meticulously optimized to a 3:1 volumetric proportion to precisely balance evaporation kinetics. Tetrahydrofuran's inherent volatility promotes rapid solidification essential for shape fidelity, while dimethylformamide's substantially higher boiling point reduces the overall solvent evaporation rate by 42 %, effectively preventing premature polymer solidification within printing nozzles—a persistent challenge in extrusion-based systems. This deliberate volatility modulation extends the critical processing window from under 15 s to over 90 s, ensuring continuous extrusion without compromise to structural integrity. Comprehensive solvent screening identified chloroform, dichloromethane, and dioxane as alternatives, but each presented significant limitations: chloroform exhibited excessive volatility causing nozzle crystallization at ambient humidity exceeding 40 %, while dioxane generated irregular morphologies due to delayed phase separation kinetics [[43], [44], [45], [46]]. Only the tetrahydrofuran-dimethylformamide combination achieved the essential trifecta of reliable extrusion, controlled fiber formation, and biocompatible residue profiles after washing, with residual solvent concentrations maintained below 50 parts per million [47].

Our study employs a phase separation method to form micron-scale fibrous rough surfaces and nanoscale micropores on material surfaces under low-temperature conditions. By adjusting the relevant parameters and conditions of phase separation, the pore size and porosity of the generated micro-nano fibrous surfaces can be controlled [48,49]. At low temperatures, the migration rate and crystallization rate of polymer components in solution increase dramatically [50]. The thermal parameters governing phase separation profoundly influence the resulting scaffold architecture. Liquid nitrogen quenching at −196 °C generates rapid ice crystal nucleation that templates expanded pore structures, whereas higher temperatures yield progressively smaller pores. This thermal control mechanism, coupled with solvent engineering, enables unprecedented precision in topographical development without toxic additives. The resultant scaffolds demonstrated exceptional biofunctionality: the 400-μm orthogonal macropores supported robust vascularization reaching 32.7 % CD31-positive vessel density—approaching native calvarial bone values (35–40 %)—while the subcellular nanofibers generated mechanical stimuli exceeding 12 nN per square micrometer. In addition, a large number of CD31-positive neovascularization signals were observed during the process of promoting bone tissue regeneration in the PLA-196 group. Combined with the verification of gene expression, we believe that PLA-196 can promote angiogenesis in the early stage of bone healing, possibly by up-regulating key hypoxia and growth factor signaling pathways. This synergistic effect of angiogenesis will greatly enhance the healing efficiency of bone tissue. It provides temporary mechanical support and guides bone tissue ingrowth during the early stages (e.g., the first 4–8 weeks), followed by a more rapid degradation rate to “create space” for the maturation and load-bearing capacity of new bone. This design aims to avoid long-term foreign body reactions and stress shielding effects. Furthermore, the lack of CD86^+^ M1 macrophage infiltration demonstrates that the scaffold degradation process did not initiate a detrimental innate immune response.

This physical cue activated microtubule-actin crosslinking factor 1-mediated mechanotransduction through phosphorylation of focal adhesion kinase at tyrosine residue 397, subsequently amplifying phosphoinositide 3-kinase/protein kinase B signaling 3.8-fold relative to smooth surfaces [30,51,52]. Consequently, expression of osteogenic transcription factors Runx2 and osterix increased 4.1-fold and 3.3-fold respectively, mechanistically providing a mechanistic explanation for the higher extent of new bone formation observed in vitro (alkaline phosphatase activity: 23.43 U/L) and in vivo (bone volume fraction reaching 62 % at eight weeks, with a densest, uniform distribution of Col-1–positive expression). At the same time, we also found that our scaffolds can significantly regulate the MACF1 gene and improve the skeletal morphology of cells to promote bone formation. Extensive evidence establishes Piezo1 as a critical mechanosensor for substrate stiffness. On stiffer surfaces like PLA-196, enhanced cell spreading and cytoskeletal tension activate Piezo1, triggering Ca^2+^ influx. This initiates downstream signaling through Ca^2+^/Calmodulin and YAP/TAZ pathways—consistent with our transcriptomic data—ultimately driving osteogenic differentiation. Furthermore, Piezo1-mediated Ca^2+^ flux promotes Akt phosphorylation, aligning with our observed enrichment of PI3K-Akt and Wnt pathways. The enhanced osteogenicity of PLA-196 stems from a mechanotransduction cascade where elevated substrate stiffness promotes Piezo1-mediated calcium influx, thereby activating downstream signaling pathways essential for bone formation. The high cell spread rate of the PLA-196 group led to a higher cell density under the microscope, yet there was no difference in its actual density compared with each group.

Degradation kinetics emerged as equally critical to functional success [[53], [54], [55]]. We have supplemented the stress-strain curve to better visualize its mechanical performance. Micro-CT results indicate that although the scaffold exhibits slight mass loss during the initial two weeks, its macroscopic structural integrity is maintained until at least the fourth week. More importantly, the mechanical performance degradation curve demonstrates that the decline in compressive modulus is gradual rather than abrupt. This process provides a crucial time window for cell migration, proliferation, and extracellular matrix deposition. This degradation profile synchronized precisely with the peak phase of new tissue formation, unlike slower-resorbing polymers such as polycaprolactone (28 % mass loss at eight weeks) where persistent fragments mechanically impede ossification. While the compressive modulus of 9.83 MPa suffices for craniofacial applications, load-bearing anatomical contexts necessitate mechanical augmentation strategies including increasing the PLA proportion in the composite, nano-hydroxyapatite blending (demonstrated to elevate modulus to 23 MPa without compromising nanotopography), or polycaprolactone reinforcement frameworks [53,54].

Transcriptome analysis revealed significant enrichment of Wnt and PI3K-Akt pathways in PLA-196 groups, providing mechanistic insight into their superior osteogenic performance. Although direct pharmacological validation remains for future study, the strong concordance between pathway enrichment and functional phenotypes supports their biological relevance. Specifically, these molecular signatures consistently align with enhanced ALP activity, osteocalcin expression, and in vivo bone formation observed in PLA-196 scaffolds. While further studies using pathway modulators are needed to establish causality, the current multi-level evidence positions Wnt and PI3K-Akt as promising mechanistic candidates, offering rational targets for designing next-generation biomaterials with optimized osteoinductive properties. Future clinical translation requires resolution of sterilization sensitivities. In conclusion, the strategic integration of solvent-engineered printing with ultrafast thermal phase separation establishes a versatile and clinically viable platform for bone regeneration scaffolds. Beyond architectural precision, this approach uniquely transduces topological information into defined cellular responses through mechanobiological pathways, effectively resolving the historical compromise between structural complexity and biological safety. The methodology establishes new possibilities for personalized bone reconstruction while providing fundamental insights into the mechanobiology of osseous regeneration. While this approach effectively demonstrates the osteoconductive potential of our low-temperature phase-separated PLA scaffolds, we recognize that future work should extend to load-bearing bone models to investigate scaffold performance under physiologically relevant mechanical demands. Such studies will be essential for translating these promising materials into clinical applications requiring structural support. Future iterations incorporating immunomodulatory microenvironments or spatiotemporal growth factor delivery could further enhance regenerative outcomes in complex clinical scenarios including osteoporotic defects and irradiated tissues.

We systematically compared the PLA-based scaffold developed in this study with current mainstream bone graft substitutes to clarify its unique positioning and value. Compared to ceramic-based materials (such as hydroxyapatite HA and β-tricalcium phosphate β-TCP), our PLA scaffold demonstrates significant advantages in matching mechanical properties [56] Ceramic materials typically exhibit high compressive strength but high brittleness, whereas natural bone is a resilient composite material. Through precise topological design, our PLA scaffold achieves a modulus closer to natural bone, effectively avoiding the “stress shielding” effect and thereby promoting mature bone remodeling. Compared to other synthetic polymer systems (such as polycaprolactone, PGA, and their copolymer poly PLGA), the PLA scaffold in this study offers more ideal degradation rate coordination. PGA degrades too rapidly, potentially compromising mechanical support; PCL degrades too slowly, potentially hindering complete bone replacement. Our scaffold demonstrated significant degradation and new bone formation within 8 weeks, indicating its degradation kinetics align more closely with the critical bone defect repair cycle. More importantly, our innovative surface topography design effectively guided cellular behavior without introducing additional bioactive factors, offering potential advantages over systems requiring complex growth factor loading (e.g., BMP-2, VEGF). Compared to strategies relying on chemical factor release, surface structural signals provide benefits such as precise spatial localization, high stability, and resistance to enzymatic degradation [57]. Our findings demonstrate that meticulously engineered microstructures can mimic the physical cues of natural bone ECM. By regulating cytoskeletal reorganization and mechanical signal transduction, these structures directly promote osteogenic differentiation and angiogenesis, providing novel experimental evidence for achieving “growth factor-free” bone regeneration strategies.

Although this study achieved encouraging results in animal models, we are clearly aware of its limitations and the challenges it faces in moving towards clinical application. Firstly, the rat skull defect model adopted in this study is a healthy one without a background of systemic diseases. However, in clinical practice, patients requiring bone transplantation often have systemic metabolic diseases such as osteoporosis and diabetes, and their local microenvironment, inflammatory state and osteogenic potential are all in a impaired state. Under these pathological conditions, whether the osteogenic efficacy of this scaffold will be compromised still needs to be verified in more complex animal models. Secondly, the long-term fate of the scaffold still needs further exploration. Whether the acidic microenvironment of PLA degradation products will cause chronic inflammatory responses over a longer time scale also requires close attention.

Challenges also exist at the manufacturing and regulatory levels. To achieve clinical transformation, it is essential to ensure the repeatability of large-scale production, including the uniformity of polymer molecular weight and nanotopological structure, as well as the precise control of 3D printing parameters. The sterilization process may have an impact on the physicochemical properties and biological performance of the stent, which needs to be evaluated in subsequent studies.

## 5 Conclusion

In this study, we developed a hybrid manufacturing strategy for fabricating polylactic acid (PLA) scaffolds by integrating extrusion-based 3D printing with low-temperature thermally induced phase separation (TIPS). By optimizing solvent ratios and freezing temperatures, scaffolds with tunable micro-nano fibrous surfaces and macroporous structures were successfully fabricated. The synergistic application of these technologies significantly promoted the uniform distribution and osteogenic differentiation of BMSCs on the scaffolds. Specifically, micro-nano fibrous surfaces with different structures were found to modulate the expression of MACF1, thereby improving cytoskeletal morphology and mediating osteogenic differentiation. Further validation in animal models demonstrated the remarkable effectiveness of the grid-structured scaffolds in promoting bone regeneration, characterized by substantial new bone formation and a significant increase in the expression of key bone repair markers. These findings confirm that the developed grid-structured scaffolds are not only theoretically innovative but also exhibit high potential and practical efficacy in enhancing bone healing for bone tissue engineering applications, thereby providing new insights and robust technical support for future research and applications in the field of bone repair.

## CRediT authorship contribution statement

**Xinyi Yun:** Writing – review & editing, Writing – original draft, Visualization, Validation, Supervision, Software, Project administration, Methodology, Investigation, Formal analysis, Data curation. **Ziyue Li:** Visualization, Validation, Supervision, Software, Resources, Methodology, Formal analysis, Data curation, Conceptualization. **Zi Yan:** Writing – original draft, Visualization, Validation, Supervision, Software, Methodology, Formal analysis, Data curation. **Shiyu Li:** Writing – review & editing, Supervision, Funding acquisition. **Zhenning Dai:** Software, Resources, Project administration, Methodology, Investigation. **Jintao Hu:** Validation, Supervision, Resources, Project administration. **Yueyi Ren:** Supervision, Software, Resources. **Liming Huang:** Project administration, Methodology, Investigation. **Qingshi Wang:** Visualization, Validation, Supervision. **Chengyu Zhang:** Visualization, Validation, Supervision. **Jianxin Li:** Funding acquisition, Formal analysis, Data curation. **Chunnuan Deng:** Visualization, Formal analysis. **Han Liu:** Writing – review & editing, Data curation, Conceptualization. **Weihan Zheng:** Writing – review & editing, Methodology, Investigation, Funding acquisition. **Chong Zhong:** Writing – review & editing, Funding acquisition, Conceptualization. **Ziqi Zhang:** Writing – review & editing, Writing – original draft, Data curation, Conceptualization.

## Declaration of competing interest

The authors declare that they have no known competing financial interests or personal relationships that could have appeared to influence the work reported in this paper.

## Data Availability

Data will be made available on request.

## References

[1] Li S., Cui Y., Liu H., Tian Y., Fan Y., Wang G., Wang J., Wu D., Wang Y. (2024). Dual-functional 3D-printed porous bioactive scaffold enhanced bone repair by promoting osteogenesis and angiogenesis. Mater. Today Bio.

[2] Bian Y., Hu T., Lv Z., Xu Y., Wang Y., Wang H., Zhu W., Feng B., Liang R., Tan C., Weng X. (2023). Bone tissue engineering for treating osteonecrosis of the femoral head. Explorations.

[3] Bai Y., Wu N., Li X., Liu Z., Li K., Jiao T., Liu F. (2025). Recent progress of 3D printed responsive scaffolds for bone repair: a review. Mater. Today Bio.

[4] Joyce M., Hodgkinson T., Lemoine M., González-Vázquez A., Kelly D.J., O'Brien F.J. (2023). Development of a 3D-printed bioabsorbable composite scaffold with mechanical properties suitable for treating large, load-bearingarticular cartilage defects. Eur. Cell. Mater..

[5] Shalchy F., Lovell C., Bhaskar A. (2020). Hierarchical porosity in additively manufactured bioengineering scaffolds: Fabrication & characterisation. J. Mech. Behav. Biomed. Mater..

[6] Mahapatra C., Kim J.J., Lee J.H., Jin G.Z., Knowles J.C., Kim H.W. (2019). Differential chondro- and osteo-stimulation in three-dimensional porous scaffolds with different topological surfaces provides a design strategy for biphasic osteochondral engineering. J. Tissue Eng..

[7] Tanzli E., Kozior T., Hajnys J., Mesicek J., Brockhagen B., Grothe T., Ehrmann A. (2024). Improved cell growth on additively manufactured Ti64 substrates with varying porosity and nanofibrous coating. Heliyon.

[8] Cho Y.S., Gwak S.J., Cho Y.S. (2021). Fabrication of polycaprolactone/nano hydroxyapatite (PCL/nHA) 3D scaffold with enhanced in vitro cell response via design for additive manufacturing (DfAM). Polymers.

[9] Petousis M., David C., Sagris D., Nasikas N.K., Papadakis V., Argyros A., Stratiotou Efstratiadis V., Gaganatsiou A., Michailidis N., Vidakis N. (2025). Reinforced PHA/CNC biocomposites in extrusion-based additive manufacturing. ACS Omega.

[10] Qian Y., Li C., Feng Q., Mao X., Yang G., Chen S., Li T., Zhou X., He C. (2024). Antibacterial bioadaptive scaffold promotes vascularized bone regeneration by synergistical action of intrinsic stimulation and immunomodulatory activity. Chem. Eng. J..

[11] Islam M., Sadaf A., Gómez M.R., Mager D., Korvink J.G., Lantada A.D. (2021). Carbon fiber/microlattice 3D hybrid architecture as multi-scale scaffold for tissue engineering. Biomater. Adv..

[12] Chan S.S.L., Heath D.E., Franks G.V. (2024). 3D printing of multi-scale porous β-tricalcium phosphate scaffolds: mechanical properties and degradation. Open Ceramics.

[13] Deore B., Sampson K.L., Lacelle T., Kredentser N., Lefebvre J., Young L.S., Hyland J., Amaya R.E., Tanha J., Malenfant P.R.L., de Haan H.W., Paquet C. (2021). Direct printing of functional 3D objects using polymerization-induced phase separation. Nat. Commun..

[14] Dong Z., Cui H., Zhang H., Wang F., Zhan X., Mayer F., Nestler B., Wegener M., Levkin P.A. (2021). 3D printing of inherently nanoporous polymers via polymerization-induced phase separation. Nat. Commun..

[15] Liu B., Hao M., Chen J., Hu X., Zhong J., Chen Y., Yu H., Weng H., Zhang Z., Du T., Peng Z. (2025). Magnesium oxide nanoparticles modulate phase separation to form trabecular-structured cryogels for bone defect repair. Mater. Today Bio.

[16] Li Z., Yan W., Zhao F., Wang H., Cheng J., Duan X., Fu X., Zhang J., Hu X., Ao Y. (2023). Regional specific tunable meniscus decellularized extracellular matrix (MdECM) reinforced bioink promotes anistropic meniscus regeneration. Chem. Eng. J..

[17] Wu J., Han Y., Fu Q., Hong Y., Li L., Cao J., Li H., Liu Y., Chen Y., Zhu J., Shao J., Fu P., Wu H., Cui D., Wang B., Zhou Y., Qian Q. (2022). Application of tissue-derived bioink for articular cartilage lesion repair. Chem. Eng. J..

[18] Maisani M., Pezzoli D., Chassande O., Mantovani D. (2017). Cellularizing hydrogel-based scaffolds to repair bone tissue: how to create a physiologically relevant micro-environment?. J. Tissue Eng..

[19] Kohli N., Sharma V., Orera A., Sawadkar P., Owji N., Frost O.G., Bailey R.J., Snow M., Knowles J.C., Blunn G.W., García-Gareta E. (2021). Pro-angiogenic and osteogenic composite scaffolds of fibrin, alginate and calcium phosphate for bone tissue engineering. J. Tissue Eng..

[20] Yang Z., Long D. (2023). Editorial: polymeric biomaterials for regenerative medicine. Front. Bioeng. Biotechnol..

[21] Zhou X., Zhou G., Junka R., Chang N., Anwar A., Wang H., Yu X. (2021). Fabrication of polylactic acid (PLA)-based porous scaffold through the combination of traditional bio-fabrication and 3D printing technology for bone regeneration. Colloids and surfaces. B, Biointerfaces.

[22] Tümer E.H., Erbil H.Y. (2021). Extrusion-based 3D printing applications of PLA composites: a review. Coatings.

[23] Salamanca E., Choy C.S., Aung L.M., Tsao T.-C., Wang P.-H., Lin W.-A., Wu Y.-F., Chang W.-J. (2023). 3D-Printed PLA scaffold with fibronectin enhances in vitro osteogenesis. Polymers.

[24] Yin C., Zhang Y., Hu L., Tian Y., Chen Z., Li D., Zhao F., Su P., Ma X., Zhang G., Miao Z., Wang L., Qian A., Xian C.J. (2018). Mechanical unloading reduces microtubule actin crosslinking factor 1 expression to inhibit β-catenin signaling and osteoblast proliferation. J. Cell. Physiol..

[25] Hu L., Su P., Li R., Yan K., Chen Z., Shang P., Qian A. (2015). Knockdown of microtubule actin crosslinking factor 1 inhibits cell proliferation in MC3T3-E1 osteoblastic cells. BMB Rep..

[26] Yin C., Tian Y., Hu L., Yu Y., Wu Z., Zhang Y., Wang X., Miao Z., Qian A. (2021). MACF1 alleviates aging-related osteoporosis via HES1. J. Cell Mol. Med..

[27] Pan C., Cheng C., Zhong S., Li S., Tan W., Yao Y. (2025). In vitro study on the promotion of osteogenic differentiation by mitochondrial-derived vesicles through activation of inflammation and reprogramming of metabolic pathways. J. Orthop. Surg. Res..

[28] Yang Y., Zheng W., Tan W., Wu X., Dai Z., Li Z., Yan Z., Ji Y., Wang Y., Su W., Zhong S., Li Y., Sun Y., Li S., Huang W. (2023). Injectable MMP1-sensitive microspheres with spatiotemporally controlled exosome release promote neovascularized bone healing. Acta Biomater..

[29] Feng J., Liu J., Wang Y., Diao J., Kuang Y., Zhao N. (2023). Beta-TCP scaffolds with rationally designed macro-micro hierarchical structure improved angio/osteo-genesis capability for bone regeneration, Journal of materials science. Materials in medicine.

[30] Wang Q., Chen Y., Ding H., Cai Y., Yuan X., Lv J., Huang J., Huang J., Zhang C., Hong Z., Li H., Huang Y., Lin J., Yuan L., Lin L., Yu S., Zhang C., Lin J., Li W., Chang C., Yang B., Zhang W., Fang X. (2025). Optogenetic activation of mechanical nociceptions to enhance implant osseointegration. Nat. Commun..

[31] Luo L., Zheng W., Li J., Chen T., Xue W., Lin T., Liu M., Yan Z., Yang J., Li J., Pu J., Wu Y., Hu K., Li S., Huang W. (2025). 3D-Printed titanium trabecular scaffolds with sustained release of hypoxia-induced exosomes for dual-mimetic bone regeneration. Advanced science (Weinheim, Baden-Wurttemberg, Germany).

[32] Wang M., Wu Y., Li G., Lin Q., Zhang W., Liu H., Su J. (2024). Articular cartilage repair biomaterials: strategies and applications, materials today. Bio.

[33] Xu Z., Omar A.M., Bartolo P. (2021). Experimental and numerical simulations of 3D-Printed polycaprolactone scaffolds for bone tissue engineering applications. Materials.

[34] Yang S., Zheng X., Qian M., Wang H., Wang F., Wei Y., Midgley A.C., He J., Tian H., Zhao Q. (2021). Nitrate-functionalized poly(ε-Caprolactone) small-diameter vascular grafts enhance vascular regeneration via sustained release of nitric oxide. Front. Bioeng. Biotechnol..

[35] Wang J., Zhou D., Li R., Sheng S., Li G., Sun Y., Wang P., Mo Y., Liu H., Chen X., Geng Z., Zhang Q., Jing Y., Bai L., Xu K., Su J. (2025). Protocol for engineering bone organoids from mesenchymal stem cells. Bioact. Mater..

[36] Zeng Y., Lou A., Zhong Z., Cai Y., Yang Y., Liang H., Lin Y., He Z., Zhou L., Zhang Z.-Y., Wang L. (2024). Timely delivery of bone marrow mesenchymal stem cells based on the inflammatory pattern of bone injury environment to promote the repair of calvarial bone defects in rats: an optimized strategy for bone tissue engineering. J. Tissue Eng..

[37] Chiara G., Letizia F., Lorenzo F., Edoardo S., Diego S., Stefano S., Eriberto B., Barbara Z. (2012). Nanostructured biomaterials for tissue engineered bone tissue reconstruction. Int. J. Mol. Sci..

[38] Tohidnezhad M., Kubo Y., Lichte P., Heigl T., Roch D., Barahmand Pour N., Bergmann C., Sönmez T.T., Hock J.V.P., Fragoulis A., Gremse F., Rosenhain S., Slowik A., Bienert M., Kweider N., Wruck C.J., Jahr H., Hildebrand F., Pape H.C., Neuß S., Fischer H., Pufe T. (2020). Effects of strontium-doped β-Tricalcium scaffold on longitudinal nuclear Factor-Kappa beta and vascular endothelial growth factor Receptor-2 promoter activities during healing in a murine critical-size bone defect model. Int. J. Mol. Sci..

[39] Inagaki M. (2023). Cell reprogramming and differentiation utilizing messenger RNA for regenerative medicine. J. Dev. Biol..

[40] Rodríguez-González R., Delgado J.Á., Delgado L.M., Pérez R.A. (2024). Silica 3D printed scaffolds as pH stimuli-responsive drug release platform. Mater. Today Bio.

[41] Sousa A.C., Biscaia S., Alvites R., Branquinho M., Lopes B., Sousa P., Valente J., Franco M., Santos J.D., Mendonça C., Atayde L., Alves N., Maurício A.C. (2022). Assessment of 3D-Printed polycaprolactone, hydroxyapatite nanoparticles and diacrylate poly(ethylene glycol) scaffolds for bone regeneration. Pharmaceutics.

[42] Wang W., Rossi R.M., Wei K. (2025). Polylactic acid green gels for fabrication of porous scaffolds. Int. J. Biol. Macromol..

[43] Jafari A., Eslami Moghadam M., Mansouri-Torshizi H. (2023). Green synthesis and bioactivity of aliphatic N-Substituted glycine derivatives. ACS Omega.

[44] Warburton L., Rubinsky B. (2023). Cryopreservation of 3D bioprinted scaffolds with temperature-controlled-cryoprinting. Gels.

[45] Khalaj R., Tabriz A.G., Junqueira L.A., Okereke M.I., Douroumis D. (2024). 3D printed stents using fused deposition modelling. J. Drug Deliv. Sci. Technol..

[46] Afshar Mogaddam M.R., Altunay N., Tuzen M., Katin K.P., Nemati M., Lotfipour F. (2021). Headspace μ–solid phase extraction of 1,4–dioxane and 2–methyl–1,3–dioxolane from shampoo samples in a home–mode device and large volume injection of deep eutectic solvent: theoretical and experimental studies. Microchem. J..

[47] Ausellé-Bosch S., Pardo M., Pareja M., Polonio-Alcalá E., Puig T. (2025). Screening of electrospun PS/PCL scaffolds for three-dimensional triple negative breast cancer cell culture: impact of solvent, hydrophobicity, and setup orientation. Sci. Rep..

[48] Sabzi E., Abbasi F., Ghaleh H. (2020). Interconnected porous nanofibrous gelatin scaffolds prepared via a combined thermally induced phase separation/particulate leaching method. J. Biomater. Sci. Polym. Ed..

[49] Roy D., Mandal S., Chandrakar K., Dwivedi M. (2025). Controlling porosity and multifunctionality in electrospun polymeric fibers by nanoscale phase separations: Flory–Huggins interaction parameters revisited. Macromol. Chem. Phys..

[50] Zhang T.-Q., Hao S., Xiao J., Jia Z.-Q. (2023). Preparation of Poly(4-methyl-1-pentene) membranes by low-temperature thermally induced phase separation. ACS Appl. Polym. Mater..

[51] Chen X., Zhi X., Wang J., Su J. (2018). RANKL signaling in bone marrow mesenchymal stem cells negatively regulates osteoblastic bone formation. Bone Res..

[52] Han R., Zhou D., Ji N., Yin Z., Wang J., Zhang Q., Zhang H., Liu J., Liu X., Liu H., Han Q., Su J. (2025). Folic acid-modified ginger-derived extracellular vesicles for targeted treatment of rheumatoid arthritis by remodeling immune microenvironment via the PI3K-AKT pathway. J. Nanobiotechnol..

[53] Bakhtiari H., Nouri A., Khakbiz M., Tolouei-Rad M. (2023). Fatigue behaviour of load-bearing polymeric bone scaffolds: a review. Acta Biomater..

[54] Zhu L., Tong X., Ye Z., Lin Z., Zhou T., Huang S., Li Y., Lin J., Wen C., Ma J. (2022). Zinc phosphate, zinc oxide, and their dual-phase coatings on pure Zn foam with good corrosion resistance, cytocompatibility, and antibacterial ability for potential biodegradable bone-implant applications. Chem. Eng. J..

[55] Jain S., Yassin M.A., Fuoco T., Liu H., Mohamed-Ahmed S., Mustafa K., Finne-Wistrand A. (2020). Engineering 3D degradable, pliable scaffolds toward adipose tissue regeneration; optimized printability, simulations and surface modification. J. Tissue Eng..

[56] Barcena A.J.R., Ravi P., Kundu S., Tappa K. (2024). Emerging biomedical and clinical applications of 3D-Printed Poly(Lactic Acid)-Based devices and delivery systems. Bioengineering (Basel, Switzerland).

[57] Farjaminejad S., Farjaminejad R., Hasani M., Garcia-Godoy F., Abdouss M., Marya A., Harsoputranto A., Jamilian A. (2024). Advances and challenges in polymer-based scaffolds for bone tissue engineering: a path towards personalized regenerative medicine. Polymers.

