# Supplementary material for: 3D printing combined with thermally induced phase separation for engineering hierarchical osteogenic PLA scaffolds
Source: Mater Today Bio. 2025 Dec 1;35:102621. doi: 10.1016/j.mtbio.2025.102621 (PMC12721073; doi:10.1016/j.mtbio.2025.102621)
Supplement: Multimedia component 1 [file mmc1.docx]

**Supplementary Information**

**3D Printing Combined with Thermally Induced Phase Separation for Engineering Hierarchical Osteogenic PLA Scaffolds**

Xinyi Yun^1,2✝^, Ziyue Li^3✝^, Zi Yan^4✝^, Shiyu Li^2,5,6✝^, Zhenning Dai^7^, Jintao Hu^4^, Yueyi Ren^8^, Liming Huang^6^, Qingshi Wang^6^, Chengyu Zhang^1^, Jianxin Li^4^, Chunnuan Deng^4^, Han Liu^5,6✉^, Weihan Zheng^9,2✉^, Chong Zhong^1✉^, Ziqi Zhang^1✉^

**Affiliations**

1 Department of Biliary-Pancreatic Surgery, State Key Laboratory of Traditional Chinese Medicine Syndrome, The First Affiliated Hospital of Guangzhou University of Chinese Medicine, Guangzhou University of Chinese Medicine, Guangzhou, 510405, China

2 Department of Immunology, Institute of Geriatric Immunology, School of Medicine, Jinan University, Guangzhou, 510632, China

3 Department of Cardiovascular Medicine, The Second Xiangya Hospital, Central South University, Changsha, 410000, China

4 Department of Urology, Sun Yat-sen Memorial Hospital, Sun Yat-sen University, Guangzhou, 510120, China

5 Institute of Translational Medicine, Shanghai University, Shanghai, 200444, China

6 Sanming Second Hospital, Sanming, 366000, China.

7 Department of Stomatology, Guangdong Provincial Key Laboratory of Research and Development in Traditional Chinese Medicine, Guangdong Second Traditional Chinese Medicine Hospital, Guangzhou, 510095, China

8 Angiitis Department of The Affiliated Traditional Chinese Medicine Hospital, Guangzhou Medical University, Guangzhou, 51006, China

9 Guangdong Medical Innovation Platform for Translation of 3D Printing Application, The Third Affiliated Hospital of Southern Medical University, Southern Medical University Guangzhou, 510630, China

✝These authors contributed equally to this work.

**Corresponding author**

[liuhanqiu@shu.edu.c](mailto:liuhanqiu@shu.edu.cn)[n](mailto:liuhanqiu@shu.edu.cn) (H. Liu)

coilyych@smu.edu.cn (W.Zheng)

zhongchong1732@gzucm.edu.cn (Z.Chong)

[nanyuanbeizhe999@163.com](mailto:nanyuanbeizhe999@163.com) (Z.Zhang)


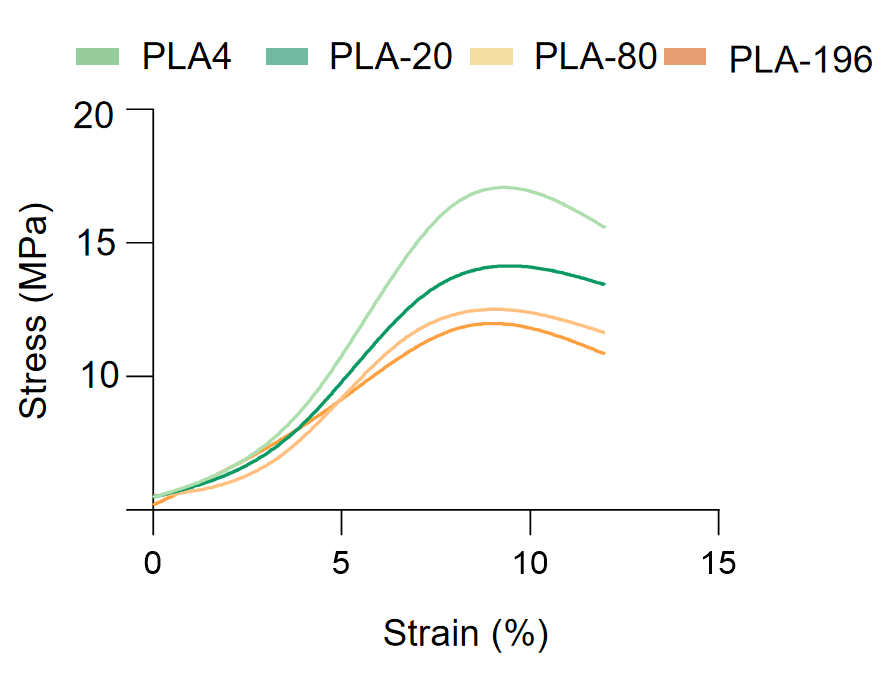


**Figure S1** Compressive stress-strain curves of PLA scaffolds.


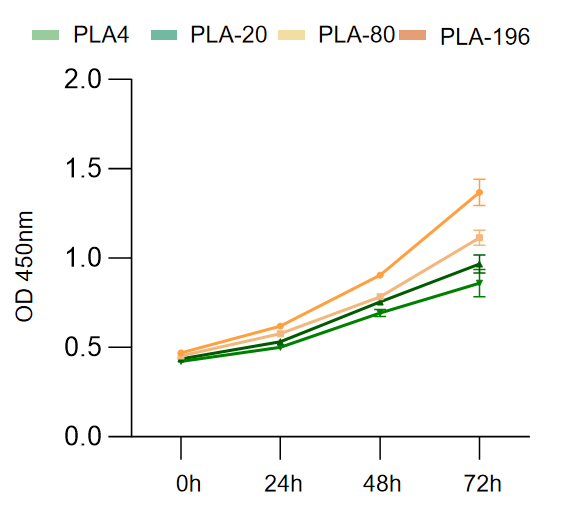


**Figure S2** CCK-8 cell viability assay of BMSCs cultured on PLA scaffolds.


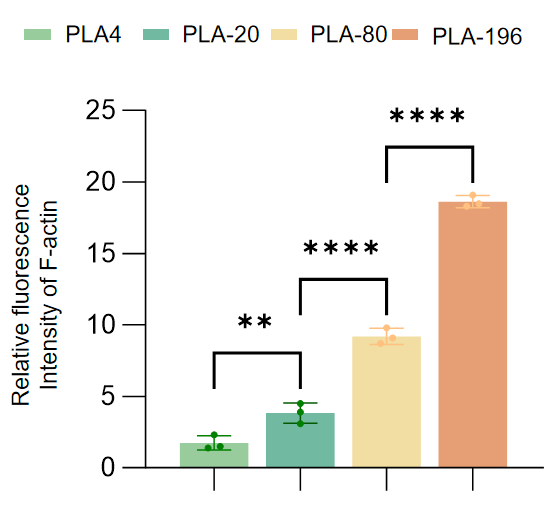


**Figure S3** Quantitative analysis of relative fluorescence intensity of F-actin in BMSCs cultured on PLA scaffolds. **: *p* < 0.01, ****: *p* < 0.0001.


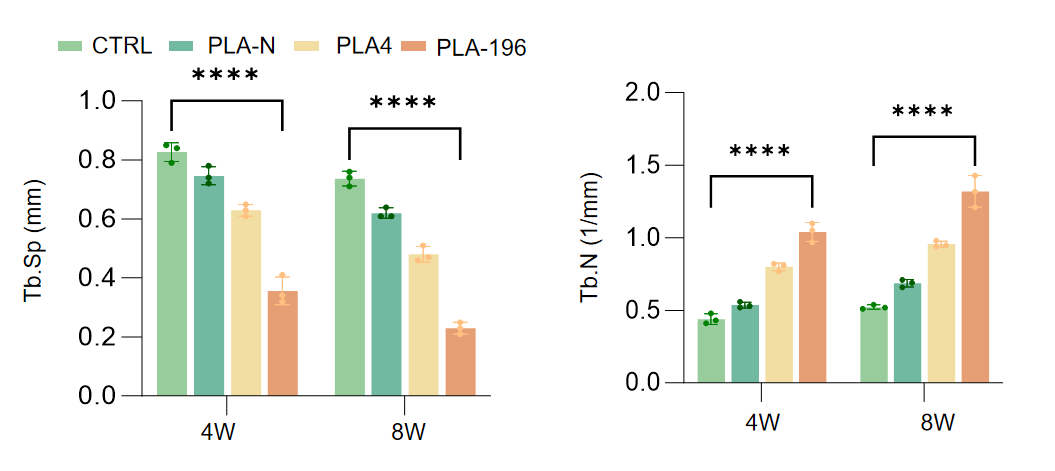


**Figure S4** (A) Trabecular separation (Tb.Sp) demonstrated corresponding decrease with improved bone quality. (B) Trabecular number (Tb.N) showed significant increase in PLA-based scaffold groups. ****: *p*< 0.0001.


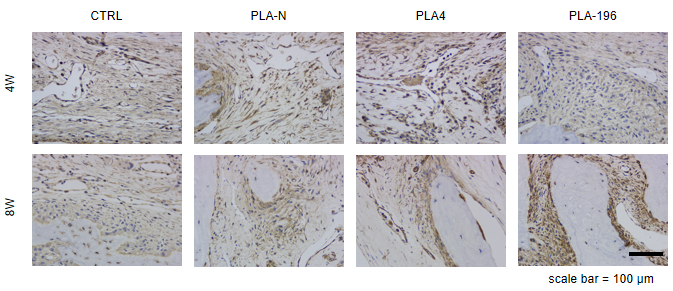


**Figure S5** Immunohistochemical staining for OCN.


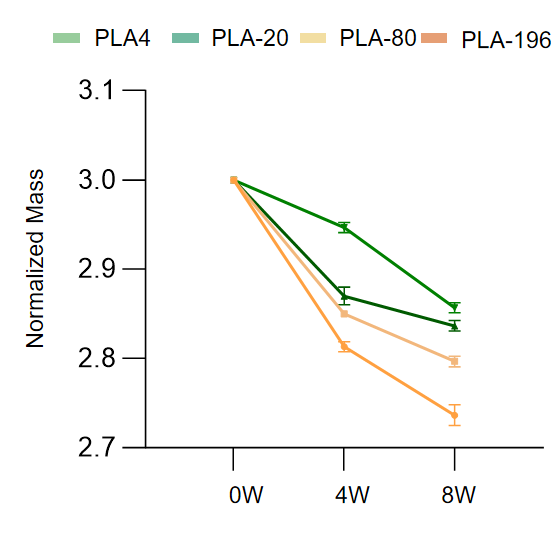


**Figure S6** Relative mass degradation profile.


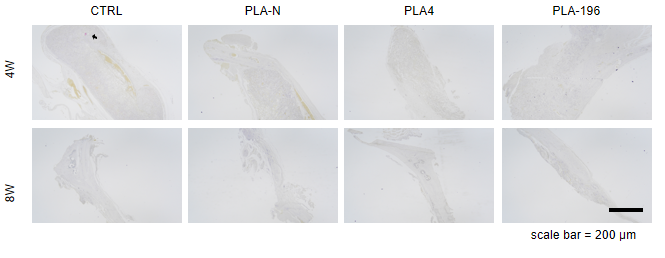


**Figure S7** TRAP staining in SD rat calvarial defect models at 4 weeks post-implantation, scale bar = 200 µm.


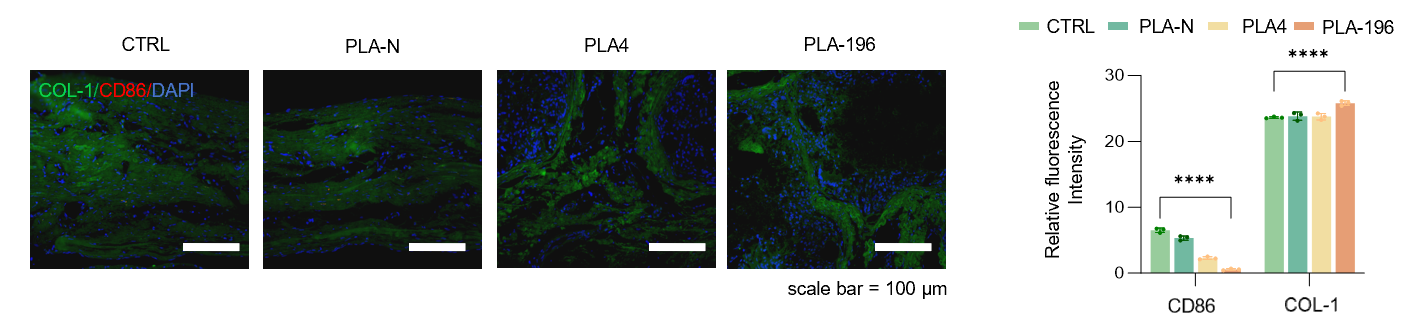


**Figure S8** Macrophage markers in SD rat calvarial defect models. (A) Immunofluorescence staining of CD86 (red) and COL-I (green) at 2 weeks post-implantation, scale bar = 100 µm. (B) Quantification of CD86 relative fluorescence intensity. ****: p< 0.0001.


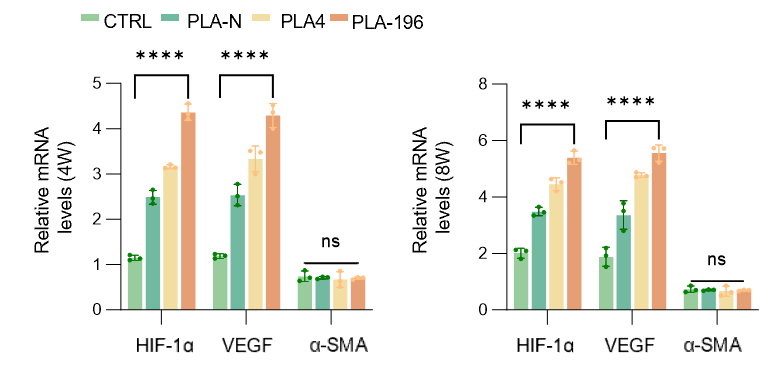


**Figure S9** qRT-PCR detection of angiogenesis related genes, ns: *p* > 0.05, ****: *p*< 0.0001.
